# The impact of anxiety upon cognition: perspectives from human threat of shock studies

**DOI:** 10.3389/fnhum.2013.00203

**Published:** 2013-05-17

**Authors:** Oliver J. Robinson, Katherine Vytal, Brian R. Cornwell, Christian Grillon

**Affiliations:** Section on the Neurobiology of Fear and Anxiety, National Institute of Mental HealthBethesda, MD, USA

**Keywords:** anxiety, cognition, threat of shock, anxiety disorders, perception, attention, learning and memory, executive function

## Abstract

Anxiety disorders constitute a sizeable worldwide health burden with profound social and economic consequences. The symptoms are wide-ranging; from hyperarousal to difficulties with concentrating. This latter effect falls under the broad category of altered cognitive performance which is the focus of this review. Specifically, we examine the interaction between anxiety and cognition focusing on the translational threat of unpredictable shock paradigm; a method previously used to characterize emotional responses and defensive mechanisms that is now emerging as valuable tool for examining the interaction between anxiety and cognition. In particular, we compare the impact of threat of shock on cognition in humans to that of pathological anxiety disorders. We highlight that both threat of shock and anxiety disorders promote mechanisms associated with harm avoidance across multiple levels of cognition (from perception to attention to learning and executive function)—a “hot” cognitive function which can be both adaptive and maladaptive depending upon the circumstances. This mechanism comes at a cost to other functions such as working memory, but leaves some functions, such as planning, unperturbed. We also highlight a number of cognitive effects that differ across anxiety disorders and threat of shock. These discrepant effects are largely seen in “cold” cognitive functions involving control mechanisms and may reveal boundaries between adaptive (e.g., response to threat) and maladaptive (e.g., pathological) anxiety. We conclude by raising a number of unresolved questions regarding the role of anxiety in cognition that may provide fruitful avenues for future research.

## Introduction

Anxiety disorders are a major worldwide health problem with sizeable psychological, social, and economic costs (Beddington et al., 2008). The impact of anxiety on cognitive function is a major contributing factor to these costs; anxiety disorders can promote a crippling focus upon negative life-events and make concentration difficult, which can lead to problems in both social and work environments. In such situations the state of anxiety can be seen as *maladaptive*. Anxiety can, however, also improve the ability to detect and avoid danger which, under the right circumstances—such as walking home alone in the dark—can be *adaptive*. The precise impact of anxiety on cognition is, however, unclear. In this narrative review we focus on an emerging, translational, within-subjects state anxiety induction method—threat of unpredictable electrical shock—which may help quantify the impact of anxiety on cognition.

## Defining cognition and anxiety

### Cognition

We define *cognition* as “information processing” (the term comes from the Latin *cognoscere*, which means “to conceptualize,” “to know,” or “to recognize”). Processing information from the outside world and determining how to use that information increases adaptive strength and reproductive success. In this review, we make broad a distinction between *hot cognition*, which involves affective (i.e., emotionally valenced) information, and *cold cognition*, which involves affectively neutral information. These categories are likely too simplistic, but they have heuristic value as a broad framework in which to dissociate effects. Across both of these cognitive categories, we also make a distinction between (1) *sensory*-*perceptual processes* (i.e., early processing and detection of stimuli); (2) *attention/control* (i.e., the ability to attend to some stimuli and ignore others); (3) *memory* (i.e., maintenance and retrieval of information); and (4) *executive function* (i.e., complex integrative and decision-making processes). These functions are presented in order of, broadly speaking, ascending phylogenetic “complexity”; perceptual processes occur rapidly, largely in subcortical and posterior cortical circuits, and attention, higher-order learning and executive processes require progressively more complex integration of cortically processed information. There are, of course, many exceptions, but these four broad divisions form the logical hierarchical structure for this review.

### Anxiety

Throughout this paper, we examine the effects of anxiety on the above cognitive functions. To this end, anxiety is defined as the response to prolonged, unpredictable threat, a response which encompasses physiological, affective, and cognitive changes (Grillon et al., 1991; Grillon, 2008; Davis et al., 2010). According to this definition, anxiety is distinct from fear; a response to acute predictable threats. Fear and anxiety are dissociable at the behavioral, neural, and pharmacological level (Grillon et al., 1991; Grillon, 2008; Davis et al., 2010). Anxiety states appear to be well-conserved across numerous species, and as such (similarly to fear), they confer adaptive value. Specifically, in unfamiliar and uncertain environments, cautious avoidance while maintaining heightened vigilance and action readiness for signs of imminent danger improves survival odds (Kalin and Shelton, 1989). However, if this behavior is adopted permanently it can become maladaptive.

In this review, we focus on functional responses evoked in healthy volunteers using the translational threat of shock paradigm, an experimental model of anxiety which operationalizes anxiety in the same manner as our definition above—as responses to prolonged unpredictable threats. These threats are non-contingent upon the task and are rare and uncontrollable.

Why do we need human models of anxiety? By examining anxiety under carefully controlled conditions, we can clarify cause-effect relations and bridge the gap between the human and animal literatures on anxiety. Although human models have important limitations (e.g., no knockout models or single cell recording), they also present the key advantage of taking into account certain features of behavior and higher-order cognition that cannot be modeled in animals (e.g., subjective, conscious experiences, or language). Human models of anxiety, such as threat of shock, do not model a pathological state but an adaptive response. As such, they provide research tools to study *functional* responses, which are a prerequisite to identifying dysfunctional mechanisms. Despite the ubiquity of anxiety, and the global burden of maladaptive anxiety (Beddington et al., 2008), our understanding of the neural, systems, and psychological mechanisms underlying anxiety-cognition interactions is surprisingly lacking.

The objectives of this review are thus two-fold, (1) to describe the effect of induced-anxiety on various cognitive processes and (2) to identify commonalities and differences with these cognitive processes in pathological anxiety and, where possible, in high dispositionally (i.e., trait) anxious individuals (see Table 1 for a thorough definition of these different types of anxiety). The guiding principle of this review is that on the one hand, where commonalities exist, the threat of shock paradigm can be used as an analog of pathological anxiety. On the other hand, when differences are identified, they may point to important boundaries between adaptive and maladaptive states.

**Table 1:** **Definitions of anxiety**.

**(A) ANXIETY DISORDERS:**
**(1) Pathological anxiety**
Pathological anxiety is associated with persistent and debilitating apprehension about negative future events, and it can have a wide range of effects on cognitive performance, including facilitative effects (e.g., threat detection) as well as detrimental effects (e.g., distractibility). Indeed, the DSM-IV definitions of anxiety disorders prominently feature “difficulty concentrating” as a key symptom. DSM-IV defines a number of different anxiety disorders including generalized anxiety disorder (GAD), phobias, panic disorder, post-traumatic stress disorder (PTSD), and obsessive–compulsive disorder [OCD; although this disorder is now thought to be more of a compulsivity disorder than an anxiety disorder (Fineberg et al., 2009)]. It should be noted that it is rare to find a patient who suffers a “pure” anxiety disorder because the rates of co-occurrence with depression are very high (Mineka et al., 1998; Kessler et al., 2012) and it is unclear how many of these symptoms can be attributed to anxiety alone.
**(B) ANXIOUS STATES:**
**(2) The threat of shock paradigm**
The threat of shock technique is a robust, translational (Davis et al., 2010), and well-validated (Schmitz and Grillon, 2012) within-subjects anxiety induction technique in which subjects are told that they are at risk of infrequent electrical shocks. Whilst anticipating the shocks subjects can be tested upon a cognitive task. This can alternate with a “safe” no shock condition to directly manipulate state anxiety *within subjects*. Such designs have a number of advantages; each subject acts as his or her own control; the psychological state of interest (i.e., anxiety) is directly manipulated; and the heterogeneities (e.g., comorbidities) of patient populations are avoided (Robinson et al., 2012b).
**(3) Self-report questionnaire measures**
Another popular approach to examining the impact of anxiety on cognition is through the use of non-diagnostic questionnaires to determine a disposition to anxiety [e.g., Spielberger trait anxiety scale (Spielberger et al., 1970)] or the BIS/BAS (“behavioral approach system behavioral avoidance inhibition system”; Carver and White, 1994) that seek to capture stable attributes of anxiety, including specific triggers (e.g., public speaking, mathematical problem solving, test-taking). Dispositional/trait anxiety scores are then correlated with task outcomes or, alternatively, a median split approach compares high and low anxious subjects. Dispositional anxiety is often viewed as a vulnerability factor in the development of psychopathology but there are multiple differences when comparing across induced and dispositional anxiety. It should be noted that neither pathological nor dispositional anxiety can be turned on and off (e.g., for memory tasks, effects on encoding, and retrieval cannot be studied separately) or studied in isolation. It is therefore important to note that their associated effects cannot be irrefutably attributed to anxiety versus another related aspect of the disorder [e.g., personality factors, cognitive diathesis (Abramson et al., 2002)]. Moreover, there are a number of statistical concerns regarding self-report approaches (Shackman et al., 2006). For example; correlational analyses are not directional, median split analyses, or other extreme-groups (categorical; e.g., upper/lower tercile or decile) approaches can result in arbitrary cut-offs. In the present text we refer to studies utilizing this methodology specifically as *dispositional anxiety* studies; in contrast with the *induced-anxiety* evoked by threat of shock.
**(4) Other state anxiety paradigms**
In addition to threat of shock, there are number of other common stress/anxiety inductions including social (speech) stressors, cold pressor tests (where the hand is submerged in cold water), and viewing anxiety-inducing movies or pictures. Although this review does not focus on these techniques, they are occasionally referenced where they illuminate differences or can help interpret results under threat of shock. One key problem with some of these manipulations (such as anxiety-inducing movies) is that they are often between-session manipulations performed once at the start of a study visit, with testing following manipulation. As such, it can be unclear whether they reveal the effects of anxiety or the recovery from a stressor (Shackman et al., 2006).

## Sensory-perceptual processing

Sensory-perceptual processing is the most basic level, and the foundation of all other cognitive processing. In this context, we define sensory-perceptual processes as the early (i.e., the most temporally immediate) processing and detection of environmental stimuli (e.g., auditory tones or discrete visual cues). We examine tasks assessing (1) early sensory processing and (2) gating of early sensory processing. These tasks largely utilize affectively neutral stimuli and hence fall into the broad category of “cold” cognitive functions. Following this, we also examine (3) emotional perception which falls under the category of hot cognitive processing. The effect of both anxiety disorders and threat of shock on such processes may illuminate, in particular, a profile of the adaptive effects of anxiety. Specifically, threat of shock studies point to enhanced sensory-perceptual processing across multiple stimulus modalities as a function of anxiety. There seems, moreover, to be a hierarchy of influence with threat of shock having increasing influence on stimuli that may more likely announce a potential threat (Table 2). These findings point to a fundamental shift whereby sensory-perceptual systems are dynamically reconfigured during anxiety states to be more sensitive to sensory perturbations. Early threat detection is adaptive because it facilitates preparation for potential danger, but it can be maladaptive when innocuous stimuli are coded as threatening and when goal-directed behavior is consistently disrupted.

**Table 2:** **Effects of anxiety on sensory-perceptual processing (arrows represent direction of effect)**.

**Domain**	**Task details**	**Threat of shock**		**Anxiety disorders**	
			**References**		**References**
Early sensory	Presentation of sounds/pictures	↑	Baas et al., 2006; Cornwell et al., 2007; Laretzaki et al., 2010; Shackman et al., 2011a	↑	Knott et al., 1994; Morgan III and Grillon, 1999; Woodward et al., 2001; Ge et al., 2011
Sensory gating	Ocular motor response to sound	↑	Davis, 1998; Grillon, 2002#	↑	Grillon, 2002#
Sensory gating	Startle attenuated by cue (PPI)	↑	Grillon and Davis, 2007; Cornwell et al., 2008	=	Grillon et al., 1996; Ludewig et al., 2002; Hoenig et al., 2005
Emotion perception	Detection of negative information	↑	Grillon and Charney, 2011; Robinson et al., 2011, 2012a	↑	Monk et al., 2006; Blair et al., 2008; Roy et al., 2008

#, =: review paper.

### Cold cognition

#### Early sensory-perceptual effects

In general, anxiety sensitizes sensory cortical systems to innocuous environmental stimuli. Supporting evidence for this claim comes predominantly from abnormalities in the mismatch negativity (MMN)-evoked response in clinically anxious and vulnerable populations. The MMN (and magnetic MMN) is elicited by passive oddball procedures in which relatively rare stimuli are embedded in an otherwise uniform sequence of stimuli (e.g., deviation in auditory tone frequency). This evoked response component, which occurs between 150 and 250 ms in the post-stimulus period, is thought to reflect preattentive change detection. Auditory MMN amplitudes have been shown to be abnormally increased in two independent samples of unmedicated patients with PTSD (Morgan III and Grillon, 1999; Ge et al., 2011) and dispositional anxiety has also been shown to positively correlate with MMN amplitudes (Hansenne et al., 2003). Consistent with these findings, an investigation of threat-induced anxiety in healthy individuals found amplified cortical responding to auditory stimulus deviance (Cornwell et al., 2007), confirming that these preattentive effects are state-related.

The evidence also suggests that anxiety-enhanced sensory-perceptual processing precedes cortical involvement. Notably, ERP studies have shown that brainstem (wave V) responses to simple auditory stimulation are increased in patients with panic disorder (Knott et al., 1994) and children with high dispositional anxiety (Woodward et al., 2001). These findings suggest that anxiety boosts auditory signaling very early (~10 ms) in the afferent pathway at the relatively primitive level of the inferior colliculus. Using threat of shock, Baas et al. (2006) demonstrated the same result of increased wave V amplitudes in healthy subjects, extending key findings from the animal literature (Maisonnette et al., 1996) to humans.

These studies of auditory processing thus illustrate a close correspondence between findings of increased sensory-perceptual responding in patient and vulnerable populations and the effects of threat of shock in healthy subjects. There are two counterexamples to note, however. Menning et al. (2008) reported *reduced* MMN responses in a small PTSD sample, but medication status was not reported; thus, the significance of this potential exception cannot be properly evaluated. From the developmental literature, Reeb-Sutherland et al. (2009) reported no MMN differences between two adolescent groups that differed in dispositional anxiety. Surprisingly, each group contained a similar proportion of individuals with current anxiety, which seems to be the more relevant factor in modulating sensory-perceptual systems and thus potentially explains the lack of a difference in MMN response. Moreover, although there is no comparable work in patient populations, evidence from the visual system also indicates that early sensory processing of neutral stimuli (within 100 ms) is augmented under threat of shock, both in terms of higher-evoked response amplitudes (Shackman et al., 2011a) and faster latencies (Laretzaki et al., 2010). Altogether, anxiety states appear to fundamentally alter central sensory pathways and profoundly shape bottom-up signaling to enhance the detection of even slight changes in the environment.

#### Sensory gating effects

Sensory gating refers to filtering mechanisms that constrain afferent signaling to allow for elaborative processing of certain stimuli. The evidence from sensory gating is mixed, but may highlight the distinction between acute and chronic effects of anxiety. Increased sensory-perceptual sensitization under anxiety may lower detection thresholds for threat stimuli, but could also overload sensory systems. One key example of sensory gating is prepulse inhibition (PPI) of the startle reflex (Grillon et al., 1991). In addition to the phylogenetically-preserved potentiation of startle responses during fear and anxiety states (Davis, 1998; Grillon, 2002), it is well-established that startle responses are diminished when a weak, non-startling stimulus (prepulse) precedes the startling stimulus by a short interval (e.g., 120 ms; Blumenthal et al., 1999). Although most clinical work has focused on schizophrenia (Braff et al., 2001), potential PPI abnormalities have been studied in clinically anxious and vulnerable populations. Reduced PPI has been documented in panic disorder (Ludewig et al., 2002), obsessive-compulsive disorder (Hoenig et al., 2005) and PTSD (Grillon et al., 1996). These patients show little or no diminution of startle reactivity when the startling stimulus is preceded shortly by a weak prepulse. Evidence from measures of dispositional anxiety is less clear, with some providing additional data of reduced PPI in high anxiety and other vulnerable populations (Corr et al., 2002; Duley et al., 2007; Franklin et al., 2009), and others reporting null results (Grillon et al., 1997; Lipschitz et al., 2005; De Pascalis et al., 2013).

Two studies investigated how PPI might be modulated during sustained threat of shock (Grillon and Davis, 2007; Cornwell et al., 2008). In stark contrast to the findings above, they reported *enhanced* PPI using various prepulse stimuli (acoustic and tactile) under threat-induced anxiety. These divergent results—that anxiety patients exhibit impaired sensory gating (decreased PPI) and healthy subjects show enhanced sensory gating (increased PPI) in a threat-induced anxiety state—deserve an explanation. We can speculate that while anxiety induced by threat of shock closely models the immediate effects of negative arousal and anticipation, it does not capture the long-term (chronic) effects of stress and worry. Threat-induced anxiety states increase PPI via facilitation of sensory-perceptual processing of weak stimuli. Sensory gating mechanisms may, however, deteriorate with persistent increased sensory-perceptual sensitization, leading to reduced PPI over the long-term.

### Hot cognition

#### Emotional perception

While threat-induced anxiety can boost sensory-perceptual processing in general, it also selectively improves the processing of extrinsically and intrinsically salient stimuli. The discussion thus far has focused on studies employing simple, innocuous stimuli to study early sensory-perceptual effects. But even there we find evidence that the relative significance of some stimuli is preserved in terms of modulating sensory-perceptual responses. For instance, an infrequent oddball stimulus boosts auditory cortical processing relative to the repetitive, standard stimulus (Cornwell et al., 2007). In this case, the relative significance of the auditory stimuli is extrinsically driven by the probability of their occurrence, as though rare (i.e., unexpected) changes in the environment are especially salient in an anticipatory anxiety state (perhaps in terms of predicting imminent danger). A similar effect of threat-induced anxiety has been observed when stimuli are made relevant by task instructions (Eason et al., 1969).

More conclusive evidence that anxiety enhances sensory-perceptual processing comes from studies that include intrinsically salient stimuli. Facial displays of emotion have been heavily-used in this regard (see e.g., Haxby et al., 2000; Phillips et al., 2003). Clinical populations show comparable biases toward aversive relative to appetitive face across behavioral and neural dimensions (Monk et al., 2006; Blair et al., 2008; Roy et al., 2008), as do individuals with increased dispositional anxiety (Cools et al., 2005; Telzer et al., 2008). However, few studies have used threat of shock to determine how state anxiety may alter facial emotion processing. Behavioral measures have been used to show that compared to facial expressions of happiness, fearful expressions are correctly identified more rapidly during threat than safe conditions (Robinson et al., 2011). A follow-up fMRI study replicated this behavioral finding and provided evidence of potential neural correlates of this effect including increased prefrontal-amygdala coupling (Robinson et al., 2012a). Similarly, research has shown that while static facial displays of fear do not alone increase startle reactivity relative to neutral emotional displays, they do so if they are presented during periods of threat of shock (Grillon and Charney, 2011). These results suggest that threat-induced anxiety can boost sensory-perceptual processing of affectively-congruent stimuli, such as fearful faces that convey the more relevant signal while anticipating shock, but the methodology (e.g., sluggish fMRI response, delayed startle onset) leaves open (in contrast to the above early sensory processing findings) the possibility that other downstream cognitive processes are influencing these biases (Pessoa, 2005). Finally, a recent study examined the impact of modulating expectancy of fearful and happy faces by pairing them with neutral cues. This task revealed that threat of shock increased responses to *unexpected* fear (but not happy) faces (i.e., prediction errors) in the striatum (Robinson et al., 2013b) indicating a bias toward detecting *novel* threats under anxiety. In some respects this can be seen as adding an affective component to the MMN stimulus deviation effect outlined above (Cornwell et al., 2007).

As a contrast to studies demonstrating enhanced processing of aversive stimuli one study (Bublatzky et al., 2010) presented negative, positive, and emotionally-neutral pictorial stimuli under threat and safe conditions, but found that only the positive pictures elicited differential electrophysiological activity across contexts. In general, however, a bias toward processing negative emotional information seems to be relatively consistent across threat of shock, anxiety disorders, and dispositional anxiety.

## Attention/control

Anxious patients suffer from debilitating intrusive thoughts and feelings as well as dysregulated attention mechanisms [e.g., distractibility, impaired concentration (Eysenck et al., 2007)]. These symptoms have been linked to attentional bias for threat. Individuals with anxiety disorders or dispositional anxiety show a proclivity to detect and process threat-related information, which interferes with performance in various attentional tasks (Bar-Haim et al., 2007). However, it has become increasingly apparent that some of these deficits may be secondary to or occur in the context of a poor ability to use attentional resources (cognitive control) to flexibly adjust attention in the face of changing environment (Derryberry and Reed, 2002; Eysenck et al., 2007). Thus, attention problems in anxiety are complex and may result from an imbalance between bottom-up stimulus-driven processing (see previous section) and top-down attention control. This section is concerned with the literature on anxiety effects on attention control and attentional bias that may contribute to attention problems and distractibility. The first section focuses on non-emotional conflict, a “cold” cognitive function, while the last section deals with two “hot” cognitive functions, attentional bias and emotional interference.

While there is increased consensus indicating that “anxiety” promotes attentional bias for threat and has a detrimental effect on control processes, a closer look at the data reveal a more complex picture. There is convergent evidence for attentional bias in anxiety disorders, dispositional anxiety, and state anxiety. However, there is no similar convergent evidence of anxiety's negative effect on control processes. In general, deficits in attentional control have been reported in clinical and dispositional anxiety, but not for anxiety induced by threat of shock. If anything, induced-anxiety is associated with better attention control (Hu et al., 2012; Robinson et al., 2012b), possibly because of improvement in the selectivity of attention (Easterbrook, 1959) (Table 3).

**Table 3:** **Effects of anxiety on attentional bias and attention control tasks (arrows represent direction of effect)**.

**Domain**	**Task details**	**Threat of shock**		**Anxiety disorders**	
			**References**		**References**
Attention bias	Emotional Stroop bias toward threat	↑	Edwards et al., 2006, 2010	↑	Bar-Haim et al., 2007#
Attention bias	Dot-probe bias toward threat	↓	Shechner et al., 2012	↑	Bar-Haim et al., 2007#
Emotional interference	Emotional interference task	↑	Cornwell et al., 2011		N/A
Emotional conflict	Emotion conflict task	=	Robinson et al., 2011	↑	Etkin et al., 2010; Etkin and Schatzberg, 2012
Conflict adaptation	Emotion conflict task	=	Robinson et al., 2011	↓	Etkin et al., 2010; Etkin and Schatzberg, 2012
Non-emotional control	Interference on Classic Stroop	=	Tecce and Happ, 1964	↑	Litz et al., 1996; Lagarde et al., 2010
		↓	Agnew and Agnew, 1963; Tecce and Happ, 1964; Hu et al., 2012		
		↓	Choi et al., 2012		

#, =: review paper.

### Cold cognition

#### Non-emotional control

Increased distractibility, attentional lapses, inability to maintain attention, poor concentration, and intrusive thoughts could be secondary to amygdala-based hyper-active threat detection mechanism (Mathews and Mackintosh, 1998). However, these maladaptive behaviors also occur in the absence of external threat, raising the possibility that anxiety is associated with a general impairment in attention control (Bishop, 2009; Shin et al., 2011; Stout et al., 2013). Perhaps the most prominent model to explain deficit in cognitive performance in anxious individuals is the dual competition framework that describes the interaction between cognition and emotion (Pessoa, 2009). The model proposes that task-irrelevant threat information competes for central processing resources with cognition, potentially impairing cognitive processes (Pessoa, 2009). An extension of this model is that tasks that require attentional resources because of conflict or interference will be more affected than tasks with little conflict or interference or tasks that rely on automatic and habitual responses, which are not affected (or potentially facilitated) (Spence and Spence, 1966). An alternative is that anxiety improves the selectivity of attention because it restricts attention to peripheral cues, facilitating tasks with restricted number of cues compared to multi-cues tasks (e.g., Easterbrook, 1959). Lastly, poor attentional control could also result from sensitized perceptual processing (see above Sensory-perceptual processing). Indeed, threat of shock has dissociable effects on information processing, facilitating early perceptual processes but impairing subsequent evaluative processing (Shackman et al., 2011a,b).

So far the literature points to diverging interference effects in clinical and dispositional anxiety compared to threat of shock-induced anxiety. Clinical and dispositional levels of anxiety are both associated with enhanced interference, a finding that is consistent with the dual competition framework. In contrast, the relatively scarce studies using threat of shock tend to find *reduced* interference, suggesting that elevated anxiety states improve the selectivity of anxiety, as suggested by Easterbrook (1959). Most of the claims of poor attentional control are based on studies with individuals scoring high on measures of dispositional anxiety (Eysenck et al., 2007; Hajcak and Foti, 2008; Bishop, 2009), rather than patients with a clinical disorder. In addition, while deficits in cognitive control brain areas have been documented in clinically and dispositionally anxious populations, these deficits do not always translate into performance deficits, perhaps because anxious individuals recruit additional processing resources (Eysenck et al., 2007). Indeed, several studies have examined the performance of anxious patients in the classic color-naming Stroop (Stroop, 1935) with mixed behavioral results as both normal and impaired performance have been reported (Litz et al., 1996; Lagarde et al., 2010; Thomaes et al., 2012). Similar results have been obtained with other measures of cognitive control. For example, obsessive compulsive disorder and generalized anxiety disorders are associated with abnormal neural signs of control monitoring, as reflected in enhanced error detection mechanisms (i.e., error-related negativity; Vaidyanathan et al., 2012), without concomitant performance impairment (Stern et al., 2010; Weinberg et al., 2012).

Several studies have examined the effect of threat of shock on Stroop [or Stroop-like tasks (Choi et al., 2012), Go/NoGo (Robinson et al., 2013a), and anti-saccade tasks (Cornwell et al., 2012b)]. While all these experiments, especially Go/NoGo and anti-saccade, require some degree of control of pre-potent responses, only Stroop examines interferences from task-irrelevant stimuli. Stroop findings are somewhat inconsistent with studies showing no specific influence of threat of shock on Stroop effect (Tecce and Happ, 1964) as well as impaired (Pallak et al., 1975; Choi et al., 2012) or improved (Hu et al., 2012) performance. This inconsistency could be attributed to procedural differences, especially among older non-computerized studies (Pallak et al., 1975; Choi et al., 2012). Recently, Choi et al. (2012) used a Stroop-like “response-conflict” task in which subjects had to identify whether a picture was a house or a building while ignoring task-irrelevant congruent and incongruent words (i.e., house, bldng) printed in the middle of the pictures. Shock anticipation impaired performance on the incongruent trials (Choi et al., 2012), which the authors interpreted in the context of the dual competition framework (Pessoa, 2009), i.e., shock threat monitoring competes for central resources adversely impacting conflict processing (Choi et al., 2012). However, these results were not confirmed in another study by the same group. In fact, Hu et al. (2012) found that shock threat *improved* performance on the classic Stroop. Because improved performance was accompanied by a general increase in reaction time to the congruent and control trials, it was suggested that the better ability to resolve the conflict was caused by the adoption of a more cautious approach, trading slower speed for better performance accuracy (Agnew and Agnew, 1963; Tecce and Happ, 1964; Hu et al., 2012). It seems that adopting cautious behavior would be adaptive when anxious because it would prevent any potential impulsive response that could have devastating consequence. However, it is unlikely that shock-induced anxiety generally leads to what could be considered a cautious pro-active behavior set (Braver, 2012). Most threat of shock studies do not report a slowdown of reaction time across a wide variety of tasks (Shackman et al., 2006; Cornwell et al., 2012b; Vytal et al., 2012; Robinson et al., 2013a,b). In fact, and consistent with some models (Spence and Spence, 1966), threat of shock *facilitates* habitual responses (Cornwell et al., 2012b).

Threat of shock-mediated performance improvement during Stroop tasks may be due to a narrowing of attention that restricts attention to peripheral distracting cues (Easterbrook, 1959). This possibility is supported by a number of studies using stressors other than shock such as ego threat, time pressure, or loud noises, which have been shown to reduce stress interference on conflict and Stroop tasks (O'Malley and Poplawsky, 1971; O'Malley and Gallas, 1977; Chajut and Algom, 2003; Booth and Sharma, 2009). This may be due to a general increased in non-specific arousal. Indeed, drugs that increased physiological arousal also reduce Stroop interference (Callaway, 1959; Kenemans et al., 1999).

Further evidence that threat of shock can facilitate attention to specific stimuli comes from a recent study of sustained attention (Robinson et al., 2013a). Vigilance or sustained attention is the ability to maintain attention and alertness during prolonged and monotonous tasks. The maintenance of attention is highly dependent on attentional control; failure of attentional control leads to attentional lapses and off-task thinking (Mcvay and Kane, 2010). Robinson et al. examined the impact of threat of shock in a task in which subjects responded to highly frequent “go” stimuli but withheld responses to very infrequent neutral “nogo” targets. Threat of shock significantly reduced errors of commission (i.e., accidentally pressing during nogo targets) while having no effect on go trials or overall reaction time. This indicates that threat of shock serves to improve response inhibition by focusing attention on the infrequent nogo targets.

If stress facilitates conflict processing, then why did threat of shock impair performance during Choi et al.'s (2012) conflict-response task and Pallak et al.'s (1975) Stroop task? One possibility is that these tasks may not have been sufficiently difficult to fully occupy attentional resources. When a task does not completely occupy attentional resources, available resources can be allocated to task-irrelevant distractors, interfering with the task at hand (Bishop, 2009; Vytal et al., 2012, see also below). Choi et al.'s task was clearly not as taxing as the Hu et al.'s task. Overall reaction time in the former task was faster and accuracy in in congruent trials was much higher than in the latter task (~740 ms/3.1% errors and 960 ms/14% errors, respectively in the safe condition). Similarly, there was little time pressure in the Pallak et al.'s study. Clearly, differences in processing loads may impact the effect of anxiety on performance, a possibility that is further discussed in the memory section below.

### Hot cognition

#### Attentional bias

Cognitive models of anxiety have been influential in postulating attentional bias operating at an early stage of information processing. Specifically, attentional bias for threat may play a prominent role in the etiology and maintenance of anxiety disorders (Mogg and Bradley, 2005; Bar-Haim et al., 2007; Cisler and Koster, 2010; Macleod and Mathews, 2012). There is substantial evidence showing that dispositionally and clinically anxious individuals exhibit an attentional bias toward or away from threat (Mogg and Bradley, 2005; Bar-Haim et al., 2007; Cisler and Koster, 2010; Macleod and Mathews, 2012), a finding that tends to be replicated by threat of shock. However, the nature, mechanisms, and contexts of these biases remain to be clarified.

Initial studies indicated attentional bias toward threat in clinical and dispositional anxiety (see Cisler and Koster, 2010). However, more recent studies have demonstrated qualitatively different types of threat biases, including preferential engagement, difficulty in disengagement, or attentional avoidance (Cisler and Koster, 2010; Sheppes et al., 2013). There is now increased effort to characterize the underlying mechanisms of these components of attentional biases as well as the information processing stage at which they occur (Bar-Haim et al., 2007; Sheppes et al., 2013). Understanding the nature of anxiety mediated bias is crucial from a theoretical and a practical viewpoint. Theoretically, it is important to understand the underlying attentional mechanisms of these biases given their potential role as vulnerability markers (Bar-Haim et al., 2007). Practically, a better understanding of these mechanisms may help improve bias modification techniques aimed at changing the selective bias to threat. Such techniques show therapeutic promise as a novel treatment for anxiety (Macleod and Mathews, 2012).

There is substantial evidence that biases are not inflexible, but are, in fact, very plastic and strongly influenced by environmental stressors (Bar-Haim et al., 2010; Wald et al., 2013). These results suggest that state anxiety is a key variable in the modulation of bias (Mathews and Sebastian, 1993; Helfinstein et al., 2008). Yet, relative to the large literature on bias in clinical and dispositional anxiety, little is known about how state anxiety impacts biases. Threat of shock may be an ideal assay to investigate bias plasticity.

The two most common procedures employed to examine attentional bias are the emotional Stroop and the dot-probe tests. The emotional Stroop, which is a variation of the classic Stroop (Stroop, 1935) interference task (see below), consists of threat-related (e.g., death) and neutral (e.g., chair) words printed in varying colors (e.g., death printed in red). The subjects' task is to name the color of the word while ignoring its semantic content. Despite their high similarity, the nature of the interference in the classic Stroop and emotional Stroop are highly different. The classic color-naming Stroop examines the conflict produced by semantic incompatibility. The interference is not caused by conflict in the emotional Stroop, but by attentional capture by the emotional stimulus (Algom, 2004; Buhle et al., 2010). The emotional Stroop has been criticized because of difficulties interpreting results in term of attentional engagement and disengagement (Cisler and Koster, 2010). The dot-probe addresses some of these limitations. In the dot-probe task, word pairs, one threat-related, and one neutral are presented briefly on the screen. Subjects are required to respond as quickly as possible to a small visual stimulus that replaces one of the words following their removal. An attentional bias toward or away from threat is revealed when subjects are faster or slower, respectively, to respond to a probe that replaces a threat word relative to neutral word. Three studies examined the influence of anxiety evoked by threat of shock using the emotional Stroop in low and high dispositional anxiety individuals (Miller and Patrick, 2000; Edwards et al., 2006, 2010). Low dispositional anxious subjects displayed no attentional bias, including when they were anticipating shocks. Edwards et al. (2006, 2010) found that while high dispositional anxious subjects had no attentional bias in the control no shock condition, color-naming of threat words was delayed during shock anticipation (Edwards et al., 2006, 2010). In contrast, in another study, high dispositional anxious subjects exhibited delayed color-naming responses to threat words in the no shock control condition, an effect that was not affected by threat of shock (Miller and Patrick, 2000). The discrepancy among these studies may be explained by the different levels of dispositional anxiety of the high dispositional anxious groups. The mean dispositional anxiety score was 20% higher in the high dispositional anxious subjects in Miller and Patrick's study (54.4) compared to the Edwards et al. (2006, 2010) studies (46 and 45, respectively). Miller and Patrick's results are consistent with the literature that has documented delayed color-naming threat words in high dispositional anxiety (Bar-Haim et al., 2007), probably because high dispositional anxiety is also associated high *state* anxiety (Macleod and Mathews, 1988). Thus, threat of shock may have failed to further increase the magnitude of the effect because of a ceiling effect. These results are consistent with the proposition that in non-clinically anxious individuals, attentional bias is an interactive function of dispositional anxiety and state anxiety due to a current stressor (Macleod and Mathews, 1988).

So far, a single study has examined the effect of threat of shock on attentional bias using the dot-probe. Shechner et al. (2012) showed that under threat of shock subjects took longer to respond to a probe that followed a threat cue compared to a neutral cue, indicating vigilance away from threat (Shechner et al., 2012). These results are consistent with evidence from naturalistic studies that have demonstrated attention *away* from threat cues during combat stimulation (Wald et al., 2011) or exposure to rocket attacks (Bar-Haim et al., 2010). The apparent contradiction in the effect of threat of shock in the emotional Stroop and the dot-probe may be due to the fact that these two tests probe different aspects of bias. The emotional Stroop may engage late control processes and the dot-probe early attentional processes (Macleod et al., 1986; Bar-Haim et al., 2007). The construct of the bias within each task is also a matter of debate. It has been argued that the emotional Stroop reflects an affective reaction (i.e., a perceptual bias as outlined above) rather than an attentional process (Algom, 2004). Similarly, it is unclear whether biases associated with the dot-probe are related to disengagement difficulties or to initial orienting (Salemink et al., 2009; Clarke et al., 2013). Note, however, that it is also possible that the data from the emotional Stroop and dot-probe tests are consistent. Interference by threat cues in the emotional Stroop may reflect effortful avoidance of processing threat cues rather than attentional capture by these cues (De Ruiter and Brosschot, 1994). According to this view, threat of shock would promote threat cue avoidance, a conclusion that is consistent with a number of studies that have shown that stress can lead to a shift of attention away from danger cues (Mathews and Sebastian, 1993; Amir et al., 1996; Chen et al., 2002; Garner et al., 2006; Helfinstein et al., 2008). It may therefore be that, consistent with the emotion perception studies highlighted above (Table 1) (Grillon and Charney, 2011; Robinson et al., 2011, 2012a), anxiety biases the processing of threats, but the different tasks assess different adaptive responses (i.e., approach or avoidance) to these threats.

Recently, Etkin et al. (2006, 2010) introduced a novel emotion conflict Stroop-like procedure. The task requires subjects to identify the expression of a face (fearful or happy) while ignoring words “happy” or “fear” superimposed on the faces (Etkin et al., 2006). This paradigm provides a measure of two important aspects of interference, conflict detection and conflict regulation. Emotional conflict detection is the classic detection of incongruence, which results in delayed responses to incongruent trials. Conflict regulation or adaptation is the improvement of these delayed responses to incongruent trials when they follow incongruent trials, suggesting activation of emotional regulatory mechanisms (Etkin et al., 2010). Investigations of these regulatory mechanisms are in their infancy but could provide useful in understanding implicit emotional regulation (Etkin et al., 2010). Generalized anxiety disorder and panic disorder are associated with impaired conflict adaptation (Chechko et al., 2009; Etkin et al., 2010; Etkin and Schatzberg, 2012). Individuals with vulnerability to anxiety disorders due to high trait anxiety or behavioral inhibition do not show such impairment (Jarcho et al., 2013; Krug and Carter, 2012). Similarly, in the only threat of shock study conducted so far, conflict adaptation was unaffected by the anticipation of shock (Robinson et al., 2011). These results suggest that poor emotional conflict adaptation may be associated with the disease process rather than being a vulnerability marker or an outcome of state anxiety.

One leading explanation for the attentional bias in anxiety is that threat-related stimuli have a special status, namely that they are prioritized and have privileged access to the amygdala. In other words, threat-related stimuli are processed automatically. This view is supported by studies showing amygdala activation to unattended (“unseen”) threat-related stimuli (Morris et al., 1999; Vuilleumier et al., 2001). Accordingly, the amygdala plays a prominent role in the pre-attentive and *automatic detection* of threat cues. However, the automaticity of amygdala processing of such cues has been questioned by several studies arguing that amygdala activation by threat cues (e.g., fearful faces) requires attentional resources (Pessoa, 2005). These studies have demonstrated that amygdala reactivity to threat-related distractors can be abolished in perceptually demanding contexts (Pessoa et al., 2005), which is, in turn, consistent with the concept that distractors cannot be processed when perceptual capacity is exhausted (Lavie et al., 2004). Thus, while the amygdala plays an important role in threat detection it may do so in concert with other structures (Cisler and Koster, 2010).

The threat of shock could be useful to investigate the boundaries between automatic and more controlled mechanisms mediating bias. It is now clear that biases are flexible and are strongly influenced by contextual stressors (Bar-Haim et al., 2010). One possibility is that the automaticity of bias is influenced by the nature of the stressor. While there are obvious advantages in requiring control processes of mild threat distractors in an innocuous environment, this may not be adaptive when danger looms. Fast and automatic capture of potential threats may then become crucial to survival. The possibility that automaticity of threat detection depends on environmental threat was tested by Cornwell et al. (2011). These authors examined the effect of threat of shock on threat bias in a paradigm previously employed to investigate amygdala activation to task-irrelevant fearful and neutral faces under low and high perceptual load (Bishop et al., 2007). The no-threat condition replicated the basic finding of greater amygdala response to fearful compared to neutral faces under low but not high perceptual load (Cornwell et al., 2011). However, consistent with the hypothesis that anxiety sensitizes threat detection, amygdala activation to fearful faces under high perceptual load was preserved during shock anticipation (Cornwell et al., 2011). These results are therefore consistent with the hypothesis that threat detection requires processing resources in innocuous contexts but become automatic in threatening environments.

It is clear that induced-anxiety biases attention, either by changing its selectivity or it sensitivity to threat, which likely has, in turn, downstream effects on cognition that can be positive or negative depending on the nature of the task at hand.

## Memory

Memory encompasses processes involved in the encoding, storage, and retrieval of information perceived and attended to in the prior sections. While there is clear evolutionary advantage to facilitating threat detection and rapid sensory responding in unpredictable environments, these changes observed in both induced (Robinson et al., 2011, 2012a) and pathological anxiety (Morgan III and Grillon, 1999; Ge et al., 2011) may actually come at the expense of goal-directed cognitive processes, which are central to both long- and short-term (working) memory. Anxiety induced by unpredictable threat of shock has a selective effect on memory that is dependent on modality (spatial or verbal), difficulty, and task type (working memory or long-term memory). Here we therefore divide memory into two broad categories; working (short-term) memory and long-term memory. The main focus is on cold memory processes, as we highlight a deficit of work examining the impact of threat of shock on hot memory. Broadly speaking, the current literature is mixed, but there is some agreement in findings across different anxiety manipulations and anxiety profiles (dispositional or clinical). In particular, the majority of findings demonstrate that spatial working memory is disrupted by anxiety disorders and long-term episodic memory (especially for negative emotional stimuli) is *enhanced*. Threat of shock induces decrements in short-term memory accuracy on par with those seen in patients, whereas other induction methods and dispositionally anxious subjects show only capacity deficits, suggesting that threat of shock is a better model of anxious pathology. In general, performance impairments are typically associated with high state anxiety as opposed to high dispositional anxiety (Hodges and Durham, 1972; Hockey et al., 1986), suggesting that the experience of anxiety may be the primary mechanism of impairment rather than susceptibility to stress (Table 4).

**Table 4:** **Effects of anxiety on memory (arrows represent direction of effect)**.

**Domain**	**Task details**	**Threat of shock**		**Anxiety disorders**	
			**References**		**References**
Short-term memory	Performance on verbal and spatial n-back performance, Sternberg, and corsi blocks test.	↓	Lavric et al., 2003; Kalisch et al., 2006; Shackman et al., 2006; Vytal et al., 2012	↓	van der Wee et al., 2003; Boldrini et al., 2005
Short-term memory	Performance on digit span, OSPAN, or reading span; reaction time on short-term memory tasks	=	Pyke and Agnew, 1963	=	Boldrini et al., 2005
Long-term memory	Performance on recall or recognition tests of words	↑	White, 1932; Chiles, 1958; Singh et al., 1979	↑^*^	McNally et al., 1989; Friedman et al., 2000; Paunovic et al., 2002

* “hot” negatively-valenced or disorder-specific material.

### Cold cognition

#### Working memory

Working memory refers to a temporary storage system that can be used to encode, rehearse, and manipulate information in mind (Postle, 2006; Jonides et al., 2008). In contrast to long-term memory, working memory refers specifically to short-term storage of information, and there is evidence from patients with cortical lesions that suggests these two types of memory rely on partially separable neural systems (Baddeley and Warrington, 1970; Vallar and Shallice, 2007). One of the most commonly used working memory tasks is the n-back paradigm (where subjects respond to successive stimuli based on whether they match the stimulus 1, 2, or 3 trials back etc.), because cognitive load or task difficulty can be parametrically modulated.

Research in patients suggests that pathological anxiety may specifically impair *spatial* short-term memory performance; patients with different anxiety disorders show deficits in spatial working memory, *but not verbal* working memory performance or verbal working memory capacity (Kizilbash et al., 2002; van der Wee et al., 2003; Boldrini et al., 2005). In contrast, dispositional anxiety is frequently associated with reduced working memory capacity but not performance, as captured by digit span measures or increased reaction time on verbal and spatial short-term memory tasks (Darke, 1988; Macleod and Donnellan, 1993; Ikeda et al., 1996; Derakshan and Eysenck, 1998; Richards et al., 2000; Ashcraft and Kirk, 2001), however see Markham and Darke (1991), Eysenck et al. (2005), Hansen et al. (2009). Together these findings suggest that baseline anxiety may have in impact on short-term memory processing efficiency but not accuracy.

Consistent with the patient research, studies examining the n-back task indicate that threat of shock disrupts both verbal (Vytal et al., 2012, 2013) and spatial short-term memory (Lavric et al., 2003; Shackman et al., 2006) but the impairment is more robust in spatial working memory (Lavric et al., 2003; Shackman et al., 2006; Vytal et al., 2013) [see Kalisch et al. (2006) for evidence indicating that threat of shock does not impair verbal 2-back performance]. This may be because working memory impairment is the result of competition for cognitive and sensory-perceptual resources. In particular, induced-anxiety may have a more robust impact on spatial working memory because the extensive neural resources shared between anxiety and spatial working memory are less susceptible to top-down attentional control than the resources shared between anxiety and verbal working memory processes. Conversely, the impact of anxiety on verbal working memory is dependent upon cognitive load; low, but not high, cognitive load verbal working memory tasks are impaired by threat of shock (Lavric et al., 2003; Shackman et al., 2006; Vytal et al., 2012, 2013). High-load verbal working memory tasks have been shown to actually *reduce* anxiety, while low-load verbal working memory tasks are *disrupted* by anxiety. Thus, there may be a more complex interaction between verbal working memory and anxiety, which may depend upon top-down control, and leads to less robust overall effects of threat of shock on verbal memory. Together, these findings are consistent with theories that emphasize the role of shared resources in accuracy impairment [e.g., the two-component model (Vytal et al., 2012, 2013), and a model based on hemispheric asymmetries (Shackman et al., 2006)]. The basic premise of such models is that anxiety garners neural resources critical to working memory, resulting in decreased accuracy.

Working memory research has, however, demonstrated that shock anticipation does not alter *all* working memory functions. Working memory capacity tests (e.g., digit span) are unaffected by anxiety induced by threat of shock (Pyke and Agnew, 1963), suggesting that threat-related working memory impairments may be specific to processes that require ongoing maintenance in the face of interference (e.g., n-back tasks where rapid succession of stimuli must be attended to, responded to, and subsequently forgotten/ignored) as opposed to intrinsic resource limitations. This is in contrast with research examining the effects of other anxiety induction methods where working memory capacity is limited by anxiety (Schoofs et al., 2008). Specifically, working memory capacity, not performance accuracy (however see Oei et al., 2006), is impaired by threatening pictures (Lindström and Bohlin, 2012), the cold pressor test (Schoofs et al., 2008; Duncko et al., 2009), and incidental changes in state anxiety (Walker and Spence, 1964; Firetto and Davey, 1971; Lapointe et al., 2013). In contrast to research using threat of shock, these findings are in line with processing efficiency theory (Eysenck and Calvo, 1992), which argues that anxious worry (1) reduces working memory processing capacity and (2) increases effort necessary to perform the task, thus increasing reaction time [although Duncko et al. (2009) found decreased reaction time under stress]. However, these findings are muddled by other studies that show state anxiety is not related to a reduction in working memory capacity [e.g., threatening movies: no effect on n-back (Fales et al., 2008; Qin et al., 2009) test anxiety: no effect on auditory verbal working memory, but impaired short-term item recall, (Vedhara et al., 2000); cold pressor test: no effect on Sternberg item recognition, (Porcelli et al., 2008)]; and their incongruence with threat of shock working memory research. With effects limited to processing efficiency (i.e., reaction time) perhaps a more robust, evocative, and translational method like threat of shock is necessary to truly model working memory impairments associated with anxiety. Further, many of these studies are subject to methodological concerns (e.g., verification of sustained emotion induction, psychometric matching of tasks to determine specificity of effects), which limit the scope of the conclusions that can be drawn from the existing body of research [see Shackman et al. (2006) for methodological considerations in the study of emotion × cognition interactions, and Vytal et al. (2012) for further articulation of these concerns]. As such, these results should be interpreted with appropriate limitations in mind.

#### Long-term memory

Some work has shown that in contrast with certain short-term memory tasks, patients with anxiety disorders are not impaired in long-term memory (Gladsjo et al., 1998; Kizilbash et al., 2002; Boldrini et al., 2005). However, examining the literature as whole, long-term memory findings in anxiety patients are mixed; anxiety patients have been shown to exhibit impairment in long-term episodic memory (Lucas et al., 1991; Asmundson et al., 1994; Cohen et al., 1996; Airaksinen et al., 2005).

Unlike the impairment seen in working memory studies, several studies suggest that long-term memory is *facilitated* by threat of shock. There is ample research to suggest that emotional arousal and the physiological responses that can accompany it (e.g., increase in glucocorticoids, epinephrine, and norepinephrine) facilitate encoding and memory consolidation processes by the release of hormones in the brainstem and basolateral amygdala (Ledoux, 1998; Cahill et al., 2003; Roozendaal et al., 2009). Hippocampal connections with the amygdala are thought to mediate this memory enhancement (Roozendaal, 2002; Roozendaal et al., 2006). In line with this: recognition of paired word associates (Chiles, 1958; Singh et al., 1979) and free recall of word lists (White, 1932) is greater when subjects are at risk of shock [but see Weymar et al. (2013) for a null finding]. From an evolutionary standpoint, it is fitting that threatening environments may lead to better declarative memory of such experiences, so that similar situations in the future can be recognized as such and avoided.

However, the relationship between anxiety and memory is anything but straightforward. It is important to note that a meta-analytic review of studies examining the effects of stress and stress hormones on memory found the opposite effect—that declarative long-term memory is impaired by stress and that this impairment is related to an increased cortisol response (Sauro et al., 2003). A closer look at these studies reveals that timing is a key component in determining the effects of anxiety on long-term memory. The timing of the stressor (e.g., encoding, post-encoding, retrieval etc.) can impact whether or not a memory trace is solidified or disrupted (Roozendaal, 2002). Specifically, an anxiety-provoking context during episodic memory *formation* is facilitative, but during *retrieval* it is detrimental. These effects however can only be isolated in long-term memory paradigms where encoding and retrieval periods are separate. Future work should seek to dissociate the effects of anxiety upon different stages of memory formation and retrieval.

Regarding other anxiety inductions, and in contrast to working memory, long-term memory studies indicate that both encoding and retrieval are disrupted by induced-anxiety. The cold pressor test has been shown to impair long-term memory encoding and retrieval of both verbal and spatial information (Kuhajda et al., 1998; Ishizuka et al., 2007). However, there are studies to suggest that these manipulations do not affect long-term memory (Wolf et al., 2001, 2002). Again, the inconsistencies observed suggest that these methods, while sometimes effective, may not be ideal for modeling anxiety-related memory impairments.

### Hot cognition

Studies examining the impact of threat of shock on affective memory tasks are lacking. Psychosocial stress has been shown to impair retrieval of emotional words (Kuhlmann et al., 2005) and event-related potential research has shown that dispositional anxiety leads to a decreased ability to filter out threatening distractors in a working memory task (Stout et al., 2013), indicating that hot cognitive processes which impact attention also impact working memory storage efficiency. In long-term memory tasks, patients with anxiety disorders generally show impairment *unless the memories are affectively negative*, in which case long-term memory may be facilitated (Friedman et al., 2000; Paunovic et al., 2002). Specifically, individuals with clinical anxiety (McNally et al., 1989; Friedman et al., 2000; Paunovic et al., 2002) or high dispositional anxiety (Mathews et al., 1989; Reidy and Richards, 1997) tend to have better recall of threatening information [but see Mogg et al. (1987) for an alternate view]. However, the recall bias observed in dispositionally anxious participants is somewhat fragile [e.g., not replicable over experiments or experimental blocks (Norton et al., 1988; Nugent and Mineka, 1994)]; suggesting that episodic memory biases in dispositional anxiety may be transient and surface only when there are strong relationships among disposition/pathology, mood, and stimuli. This is somewhat inconsistent with the long-term memory facilitation for *neutral* stimuli under threat of shock. One possible explanation is the emotional state of subjects during encoding; in healthy subjects, when anxiety is induced or emotionally-arousing stimuli encountered, episodic memory encoding, and consolidation is enhanced, however in clinical populations, this enhancement is tied to anxiety-relevant stimuli. A large body of research demonstrates an attentional and perceptual bias toward threatening information in anxious individuals [except for PTSD where evidence for a threat bias is mixed (Buckley et al., 2000)] see previous sections; Bar-Haim et al. (2007). When anxiety is induced by threat of shock, all information is contextually linked to the anxious state and hence preferentially processed (maintained or encoded). By contrast, in anxiety disorders only negative stimuli are anxiety-relevant and so encoding may be restricted to these stimuli. In general, however, the lack of threat of shock studies in this area makes conclusions premature and future work is needed to dissociate the causes of this discrepancy.

## Executive function

Broadly speaking, we define “executive” functions as those which require *combining* information processed by the mechanisms previously reviewed. Aspects of learning, perception, and attentional control, both hot and cold, are all integrated to guide complex future-oriented behaviors. In reviewing the literature on this final cognitive domain, we focus on three types of executive function: (1) decision-making behavior, (2) planning, and (3) spatial navigation. Given the integrative nature of these functions we do not make a distinction between hot and cold processes. We show threat of shock mimics, at least in part, the effects of anxiety disorders on both planning (i.e., no effect) and decision-making (i.e., promoting harm avoidant decisions), while at the same time having the opposite effect upon spatial navigation (Table 6).

### Decision-making

There is evidence that both translational threat of shock and anxiety disorders promote harm avoidant, loss aversion, decision-making. Decision-making behavior can become more cautious and conservative under anxiety [see Starcke and Brand (2012) for a review examine a broader range of “stress” manipulations]. On the one hand, anxiety induced by threat of shock has been shown to induce premature responding (before all options are presented) in decisions where options are revealed sequentially (Keinan, 1987), but the opposite pattern (increased response time) is seen when subjects are asked to make decisions on a trial to trial basis (e.g., matching on card sorting tasks; Murphy, 1959). Moreover, in gambling tasks where probabilities are known, threat of shock can increase risk-avoidant decision-making and lead to more conservative gambles (Clark et al., 2012). This latter effect is consistent with loss aversion [alongside indifference to rewards (Shankman et al., 2012)] which has been shown in pathological anxiety disorders (Mueller et al., 2010). The same conservative style is also seen following the cold pressor test (Mather et al., 2009) [especially in female subjects (Lighthall et al., 2009, 2012)] although it may depend upon whether decisions are being made to increase gains or minimize losses. Specifically, the “reflection effect”—the tendency of individuals to make risky decisions in the loss domain but conservative decisions in the gain domain—is increased by anxiety induced via the cold pressor test (Porcelli and Delgado, 2009).

Dispositional anxiety and speech anxiety inductions, however, demonstrate the opposite effect. *Reduced* risk avoidance has been shown following speech stressors (Starcke et al., 2008); but this effect seems to be gender-dependent, with slightly improved decision-making (i.e., increased gains on Iowa gamble task) seen in anxious females and impaired decision-making restricted to anxious males (Preston et al., 2007; van den Bos et al., 2009). Similarly, high dispositional anxiety is associated with *impaired* performance on the Iowa gambling task (Miu et al., 2008) [which is also gender-dependent (de Visser et al., 2010)], and problem gamblers with high dispositional anxiety demonstrate more severe pathological gambling problems (Ste-Marie et al., 2002) [anxiety disorders can also be comorbid with problem gambling (Petry et al., 2005)]. In general, firm conclusions are premature, but there is evidence that both translational threat of shock and anxiety disorders promote harm avoidant, loss averse, decision-making while dispositional anxiety and speech anxiety inductions promote the opposite pattern.

### Spatial navigation

Spatial navigation in anxiety has been assessed via virtual reality maze tasks as well as simple pen and paper “trail-making” tasks. Note that, as a caveat, although we define this as an executive function which requires integration of multiple facets of cognition, there is an extensive literature in rodents which links aspects of spatial navigation to reflexive responding in the hippocampus (Ekstrom et al., 2003). Threat of shock in healthy individuals has been shown to enhance spatial navigation (Cornwell et al., 2012a), as has the cold pressor anxiety manipulation (Duncko et al., 2007) [with null effect of speech stressors (Starcke et al., 2008)]. In anxiety disorders, however, the opposite effect is seen; spatial navigation is impaired (Cohen et al., 1996; Mueller et al., 2009). This discrepancy could possibly be driven by the *context* of the anxiety. Anxiety may prioritize fast and easy navigation away from threats, but impair navigation which is unrelated to threats. In healthy individuals undergoing anxiety induction, the anxiety and task are contextually linked, whereas in a person with an anxiety disorder, the task is unrelated to their anxiety. As such, task-driven anxiety may improve performance while task-unrelated anxiety impairs performance. Another possibility is that there is a key difference between the “adaptive” anxious state triggered by acute anxiety inductions and the pathological, more trait-related anxiety in anxiety disorders (discussed in more detail in the Discussion section below). Acute state anxiety may improve navigation; chronic trait anxiety may impair navigation. Regardless, translational anxiety inductions and anxiety disorders seem to have opposite effects upon spatial navigation.

### Planning

Finally, planning ability can be assessed by the Tower of London task (and its variants; e.g., the Tower of Hanoi or the Stockings of Cambridge) in which subjects have to work out how many moves are required to make two patterns look identical. Threat of shock has no effect upon the one touch tower of London (Table 5; previously unpublished data using this task, see Appendix for trial example). This is, in fact, consistent with the effects of pathological anxiety disorders, which also leave planning ability intact (van Tol et al., 2011) [in contrast with depression (Elliott et al., 1997) and sad mood induction (Robinson and Sahakian, 2009) which both impair planning on this task]. This null finding (and dissociation from sad/depressed mood) provides important context, because it demonstrates that threat of shock does not have broad indiscriminate effects on executive function; it can increase risk avoidance and improve spatial navigation while leaving planning performance intact.

**Table 5:** **Impact of threat of shock on accuracy (Acc) and planning time (RT; ms) on the one touch tower of London task**.

	**Safe**				**Threat**			
	**2 move**	**3 move**	**4 move**	**5 move**	**2 move**	**3 move**	**4 move**	**5 move**
Mean Acc	0.72	0.66	0.49	0.28	0.73	0.70	0.55	0.32
s.e.m.	0.07	0.07	0.06	0.04	0.07	0.06	0.05	0.04
Mean RT	4784	5762	6231	7214	4696	5447	6344	6892
s.e.m.	231	250	207	224	253	230	247	291

**Figure A1:**
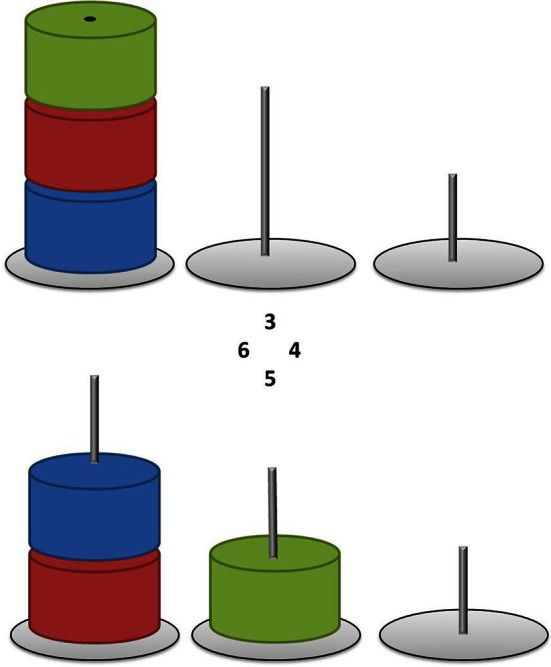
**An example stimulus from the adapted version of the tower of London—this stimulus represents a 5 move problem.** Problems are presented under both safe and threat conditions.

Twenty two healthy subjects (per structured clinical interview completed by a physician; e.g., Robinson et al., 2013a) underwent a shock work-up procedure (e.g., Robinson et al., 2011) and then completed the task under alternating threat and safe conditions (order counterbalanced). The task was adapted from previously published studies (e.g., Elliott et al., 1997; Murphy et al., 1999, 2002) and involved intermixed 2, 3, 4, and 5 move problems (see Figure A1 for an example). Subjects completed a short 5 trial practice session before commencing the task. There were a total of 6 different problems within each difficulty level. During each trial subjects were provided with 4 options for the number of moves required. Response reaction time and accuracy data were analyzed in 2 × 4 threat (safe, threat) × load (2, 3, 4, 5 move) ANOVAs. Across safe and threat conditions, task performance deteriorates as load increases [RT = F_(3, 19)_ = 42, p < 0.001; Acc = F_(3, 19)_ = 17, p < 0.001] but this does not interact with anxiety induced by threat of shock [RT = F_(3, 19)_ = 0.7, p = 0.6; Acc = F_(3, 19)_ = 0.2, p = 0.9].

**Table 6:** **Effects of anxiety on executive functions (arrows represent direction of effect)**.

**Domain**	**Task details**	**Threat of shock**		**Anxiety disorders**	
			**References**		**References**
Decision-making	Risk avoidance on gamble tasks	↑	Clark et al., 2012	↑	Mueller et al., 2010
Spatial navigation	VR maze navigation performance	↑	Cornwell et al., 2012a	↓	Cohen et al., 1996; Mueller et al., 2009
Planning	Calculate moves on tower of London	=	Table 5	=	Elliott et al., 1997; van Tol et al., 2011

## Discussion

While the threat of shock paradigm has been used extensively to examine emotional responses and defensive mechanisms (Davis et al., 2010), it is also emerging as a powerful tool to study the effects of anxiety on cognition. Below, we summarize the findings of this review before addressing questions for future research.

## The impact of threat of shock on cognition

Threat of shock facilitates early sensory-perceptual processing of neutral stimuli, improves the detection of negative information, impairs performance on tasks with emotional distractors, and facilitates resolution of conflict. In addition, threat of shock impairs short-term memory but facilitates long-term memory as well as certain aspects of decision-making and executive function. In general, the changes can be seen as part of an overall adaptive mechanism of harm avoidance in which threatening stimuli are privileged at all levels of cognitive function, but at a potential cost for some functions (e.g., short-term memory).

Thus, anxiety boosts sensory-perceptual processing, which subsequently influences downstream stages of information processing. These effects may be facilitative or detrimental depending on task demands. Consistent with the dual-model process theory (Pessoa, 2009) and attentional control theory (Eysenck et al., 2007), threat of shock affects the balance between stimulus-driven and goal-directed behaviors (Shackman et al., 2011a; Cornwell et al., 2012b), such that performance is improved when emotional information is task-relevant but impaired when it is task-irrelevant.

It is generally assumed that anxiety induces an impairment in inhibitory control, (Derryberry and Reed, 2002; Eysenck et al., 2007), which comprises the ability to inhibit prepotent responses and to resist interference from distractors (Friedman and Miyake, 2004). These two types of inhibitory control have been traditionally tested with the classic Stroop, a test of inhibition of prepotent responses, and the emotional Stroop, a test of interference by an emotional distractor. Threat of shock does not have a uniformly detrimental effect on these two tests. In fact, threat of shock impairs performance on the emotional Stroop, but, inconsistent with theoretical assumption, it *improves* performance on the classic Stroop as well as on a measure of response inhibition (Robinson et al., 2013a). There is no simple explanation for these divergent effects, which may have multiple causes, including non-specific effects [e.g., tasks not psychometrically matched (Thomaes et al., 2012)]. A critical distinction between these two tests, however, is that one is a measure of conflict and the other is not. Specifically, the classic Stroop is a true test of conflict between two responses (or inhibitory control), whereas the emotional Stroop is perhaps better characterized a measure of attentional bias (Buhle et al., 2010; Etkin et al., 2011). This suggests that the threat of shock facilitates inhibitory control, a result consistent with findings using sustained attention tasks (Robinson et al., 2013a), while at the same time increasing perceptual processing of affectively negative information. These results could, nevertheless, also be explained by other mechanisms. For example, anxiety could have opposite effect on regions of the anterior cingulate or prefrontal cortex that are differently affected by affective and non-affective incongruency (Haas et al., 2006). Anxiety also improves the selectivity of attention (Easterbrook, 1959), which could facilitate a narrowing of attention to the target during the classic Stroop. The emotional Stroop would not benefit from this selectivity because emotional distractors may be processed implicitly by the amygdala.

The differential effect of threat of shock on short-term vs. long-term memory is also of note and might be attributed to (1) to the overlap in neural resources between anxiety and short-term memory and (2) the protracted role that stress hormones play in consolidation. Short-term memory (Cohen et al., 1997) and anxiety (Etkin, 2010) both engage prefrontal mechanisms, and competition for this neural circuitry may result in temporary *impairment* due to disrupted *maintenance* of information. In contrast, episodic information *encoding* may be *facilitated* by threat of shock with the release of stress hormones in the amygdala and brainstem that serve to modulate long-term storage via the hippocampus (Cahill and McGaugh, 1998).

The impact of threat of shock on more complex executive processes such as decision-making processes can also be seen as consistent with a model of anxiety promoting cautious harm avoidance including risk-avoidant decision-making (Clark et al., 2012) and improved spatial navigation (Cornwell et al., 2012a). However, this domain of cognition is also notable for the relative paucity of studies and so considerable further work is needed to specify the precise effects.

## Differences across threat of shock and anxiety disorders

Threat of shock may thus accurately model the impact of anxiety disorders on hot cognition. One critical observation is that anxiety disorders and threat of shock have discrepant effects on (1) PPI, (2) classic Stroop, (3) conflict adaptation, (4) short-term memory capacity, and (5) spatial navigation. These unique effects in anxiety disorders are largely in cold cognitive functions which require some form of cognitive control, and are consistent with models (e.g., Bishop, 2007) postulating that anxiety disorders are associated with poor attention control. The disorder-specific effects may reflect long-term changes in response to prolonged stress or dispositional anxiety. Specifically, there may be a true dysfunctional vulnerability linked to cold function (impaired attention control; Litz et al., 1996; Lagarde et al., 2010) which either (1) predisposes vulnerable people to experience sustained affective biases which lead to a vicious cycle toward anxiety disorders; or (2) makes people anxious, which then lead to affective biases. By contrast, hot cognitive functions, including those that require cognitive control seem to be consistent across threat of shock and anxiety disorders (although it should be noted that this may vary across different anxiety disorder diagnoses). A further possibility is that threat of shock accurately induces *state* effects of anxiety, but sufferers are not always in a state of elevated anxiety and so the discrepancies across threat of shock and anxiety disorders reveal a distinction between state and trait effects. One final possibility is that discrepancies are due to the traditional inverted-U relationship between anxiety and performance. However, this seems unlikely. Many subjects experience very high levels of anxiety during threat of shock, probably higher levels relative to anxious patients tested in the laboratory (without threat of shock). In fact, one of the advantages of the threat of shock is that we can compare performance across a number of tasks keeping the level of anxiety constant. Clarifying the causes of these divergent effects is a key question for future research (see below).

## Neural mechanisms

A comprehensive understanding of the neural mechanisms underlying these effects is beyond the scope of this review. However, it is worth highlighting some recent advances pointing toward circuitry which may be involved. Both anxiety disorders and threat of shock are strongly implicated in activity in the (a) amygdala and (b) dorsal medial prefrontal cortex/dorsal anterior cingulate cortex (Shin et al., 2005; Etkin and Wager, 2007; Shin and Liberzon, 2009; Hartley and Phelps, 2012; Linnman et al., 2012; Maier et al., 2012; Robinson et al., 2012a). In fact, a circuit between these two regions is thought to drive a bias toward aversive information (Robinson et al., 2012a). This is consistent with the idea that the dorsal prefrontal (encompassing cingulate and dorsomedial) cortex is involved a emotional processing (Etkin et al., 2011), especially appraisal (Maier et al., 2012) negative affect and cognitive control (Shackman et al., 2011b). As such, it is possible that anxiety engages this circuit which then underlies some improvements in cognitive functions (both hot and cold) which promote the avoidance of danger. If turned on excessively, however, this circuit may lead to the pathological biases in anxiety disorders.

By contrast, functions which are down-regulated in anxiety share some of this neural real estate. Working memory and neutral cognitive control are both adversely affected by anxiety and are thought to be processed within regions overlapping this circuit (Pessoa et al., 2002; Shackman et al., 2011b). One possibility, therefore is that resource “overload” occurs when neural real estate critical for the aforementioned harm avoidance processes overlaps with circuitry involved in anxiety-*unrelated* processes. The preferential processing of threat avoidant stimuli may thus come at the expense of threat-unrelated processes (e.g. working memory). Cognitive functions, like planning, which are unperturbed by either threat of shock or anxiety disorders may, moreover, rely on entirely separate circuitry (Shackman et al., 2006). Of course this is likely an oversimplification, and it is worth noting that a large number of regions are implicated in anxiety, including some brain stem areas highlighted above. Going forward, the threat of shock paradigm may prove a promising tool to clarify some of these neural mechanisms.

## Future questions

Taken together, the above findings highlight a number of broad questions that might be tackled in future research:

### What causes the shift from adaptive to maladaptive anxiety?

As indicated above, there were a number of discrepant effects across threat of shock and anxiety disorders, largely on cold cognitive functions. An important question, therefore, is what drives the difference between the effect of induced-anxiety and anxiety disorders on cold cognitive functions? One possibility is that this discrepancy reveals the differences between *adaptive* and *maladaptive* anxiety. Specifically, in a threat of shock experiment, the state of anxiety is entirely rational and an adaptive response to an imminent threat. Anxiety disorders are, however, characterized by anxiety at inappropriate times or to mildly aversive stimuli; so called maladaptive anxiety. It will be important to identify the mechanism(s) by which adaptive responding becomes maladaptive. One possibility is that the same processes underlie both effects, but in the case of maladaptive anxiety, the circuitry gets “stuck” in the anxious state. This causes a broader array of stimuli to constitute threats (stimulus generalization) and impairs the ability to down regulate threats (Lissek et al., 2009; Shackman et al., 2009). What causes this switch to occur? Is there a ratcheting effect whereby once the system is pushed too far it is unable to restore healthy function? And once this occurs is that what leads to the “cold” control impairments which appear to be restricted to anxiety disorders? Clarifying the causes of these differences may reveal important mechanisms of relevance to the development of anxiety disorders.

### Can we use this understanding to improve diagnosis and treatment efficacy in anxiety?

Clarifying the impact of anxiety on cognition may allow us to more accurately assess the efficacy of treatments (Harmer et al., 2011). For instance, a potential use of threat of shock in healthy volunteers is as an analog model to identify the underlying mechanisms of these affective components in anxiety disorders. Assuming that the same mechanisms that are responsible for the effect of anxiety evoked by threat of shock on hot functions are also implicated in anxiety disorders, we can use the impacts of threat of shock on cognition in healthy individuals to screen candidate anxiolytic compounds. A serious impediment to bringing candidate anxiolytics to the marketplace is the lack of effective models to screen drugs (Rodgers, 1997; Kola and Landis, 2004; Dawson and Goodwin, 2005). This is because compounds that have anti-anxiety profile in animal models subsequently lack clinical efficacy in patients. Thus, developing a model for evaluating efficacy in humans could facilitate the screening process and bridge the gap between basic drug development and the psychopharmacological treatment of patient. Threat of shock, which appears to be a closer analog to pathological anxiety than some other anxiety inductions based upon the evidence reviewed above (e.g., Starcke et al., 2008 vs. Clark et al., 2012), could be such a model.

### Can we use cognitive interventions to treat anxiety?

On the other hand, clarifying the impact of performing cognitive tasks on anxiety, may allow us to better understand and refine cognitive treatments for anxiety. In particular, at least one threat of shock study reviewed above showed that high cognitive load serves to distract *away from* the state of anxiety (Vytal et al., 2012). Specifically, performance of a “cold” verbal n-back task reduced psychophysiological concomitants of anxiety but only under the highest (3-back) load conditions. Future work might explore whether this observation has therapeutic value. In addition, recent advances have begun to use “hot” cognitive training tasks to shift the negative biases in anxiety. In such “cognitive training” tasks, a subject's attention is implicitly shifted toward positive (at the expense of negative) emotional cues, which over time leads to reduced negative biases when assessed on cognitive tasks (Browning et al., 2010, 2012; Hakamata et al., 2010; Macleod and Mathews, 2012). This technique may eventually be used to reduce the debilitating negative biases, thus reducing anxious mood in anxiety disorders. Either way, it may be possible to adopt both hot and cold cognitive interventions to reduce the symptoms of anxiety disorders.

## Future challenges

A key advantage of threat of shock is that it provides a well-controlled manipulation of state anxiety in a within-subject design. It may help address many fundamental questions concerning the components and underlying mechanisms control, bias and regulation mechanisms at different processing stages, the role of context, and factors that contribute to inter-individual differences in bias. However, there is no single standardized “threat of shock” paradigm across the majority the reviewed studies and a clearer picture may be achievable if more variables (e.g., block-length, shock frequency) were held consistent across studies and key methodological considerations were taken into account [see, for instance the “4 methodological desiderata” in Shackman et al. (2006)]. Similarly, many investigators use the word “anxiety” without being specific about what they are talking about. Anxiety can refer to anxiety disorders, dispositional anxiety, or state anxiety (experimentally-induced anxiety); we recommend that investigators be more specific going forward.

A number of further caveats are also worth considering. First, given the number of cognitive processes there are very few studies utilizing threat of shock, leaving a large number of gaps in the literature to get a good picture of the effect of induced-anxiety on cognition. In addition, many tasks have yet to be comprehensively tested across anxiety disorders and threat of shock. Second, some threat of shock effects could reflect non-specific increased in arousal rather than specific effects due to negative affective states. However, many of the effects were selective, promoting the processing of threat- or potential threat-relevant stimuli as opposed to neutral stimuli (Mogg and Bradley, 2005; Bar-Haim et al., 2007) or stimulus-relevant as opposed to stimulus-irrelevant stimuli (Eason et al., 1969). Third, it is possible that subjects who participate in a threat of shock experiment are representative of a uniquely harm avoidant population; high dispositional anxiety subjects or subjects afraid of shock may not be inclined to participate in such experiments [although it should be noted that some researchers have used high dispositional anxiety subjects under threat of shock (Miller and Patrick, 2000; Edwards et al., 2006, 2010)]. Finally, although very few shocks are administered in threat of shock studies, it is possible that some of the effects observed were due to sensitization mechanisms (Richardson, 2000).

Future work should aim to rule-out and control for some of these potential confounds. As a general prescription going forward, we recommend that future studies should primarily (1) further investigate basic “cold” control mechanisms to lay a strong foundation for the study of “hot” cognition, (2) adopt recently developed procedures to isolate the various components of threat-induced bias, such as visual search, spatial cuing, eye tracking, and classical conditioning (Cisler and Koster, 2010; Sheppes et al., 2013; see also Clarke et al., 2013), (3) examine the interactions between (shock-induced) state anxiety, temperamental disposition (e.g., trait anxiety) and experiential factors (e.g., adverse life-events), (4) explore contextual-mediated shift in bias (Bar-Haim et al., 2010), e.g., caused by changes in shock predictability or shock temporal proximity, and (5) extend research on threat of shock to individuals with clinical anxiety. Indeed, one possibility is that specific cognitive deficits in anxious individuals may be latent and emerge only in stressful situations. Relatedly, one may ask to which extend attentional control deficits and bias are related to specific anxiety disorders or to proposed nosological distinction (i.e., fear disorders vs. distress/misery disorders; Vaidyanathan et al., 2012).

## General conclusions

In sum, we have presented an overview of the impacts of anxiety on cognition. Both threat of shock—a translational anxiety induction—and pathological anxiety disorders promote the detection of potentially harmful stimuli at multiple levels of cognition from perception to attention to memory and executive function. At the most basic level this tends to be associated with improved perception of environmental changes irrespective of valence, but at more complex levels of cognition, leads to promotion of cognitive processes relevant to harm avoidance at a cost to certain functions such as working memory, while leaving still further processes (such as planning) unperturbed. However, we also draw attention to a number of processes, such as spatial learning, PPI and non-emotional Stroop which are discrepant across threat of shock and anxiety disorders. We argue that this discrepancy, largely in cold cognitive functions, may reveal the differences between adaptive and maladaptive anxiety. Future work should attempt to delineate the causes of these differences, as well as explore the possible use of (1) cognitive interventions for the treatment of anxiety and (2) the use of threat of shock as an analog screen for candidate anxiolytics. The precise neural mechanisms underlying these effects are far from clear; this review, which is the first to collate the growing number of studies using the translational threat of shock paradigm, aims to highlight the value of this paradigm as a means to clarify these neural mechanisms. Given the large burden represented by anxiety disorders, such research is of pressing concern.

### Conflict of interest statement

The authors declare that the research was conducted in the absence of any commercial or financial relationships that could be construed as a potential conflict of interest.

## References

[B1] Abramson L. Y. Alloy L. B. Hankin B. L. Haeffel G. J. Maccoon D. G. Gibb B. E. (2002). Cognitive vulnerability-stress models of depression in a self-regulatory and psychobiological context, in Handbook of depression, eds Gotlib I. H. Hammen C. L. (New York, NY: Guilford Press), 268–294

[B2] Agnew N. Agnew M. (1963). Drive level effects on tasks of narrow and broad attention. Q. J. Exp. Psychol. A 15, 58–62

[B3] Airaksinen E. Larsson M. Forsell Y. (2005). Neuropsychological functions in anxiety disorders in population-based samples: evidence of episodic memory dysfunction. J. Psychiatr. Res. 39, 207–214 10.1016/j.jpsychires.2004.06.001 15589570

[B4] Algom D. (2004). A rational look at the emotional stroop phenomenon: a generic slowdown, not a stroop effect. J. Exp. Psychol. Gen. 133, 323–338 10.1037/0096-3445.133.3.323 15355142

[B5] Amir N. McNally R. J. Riemann B. C. Burns J. Lorenz M. Mullen J. T. (1996). Suppression of the emotional Stroop effect by increased anxiety in patients with social phobia. Behav. Res. Ther. 34, 945–948 8990547 10.1016/s0005-7967(96)00054-x

[B6] Ashcraft M. H. Kirk E. P. (2001). The relationships among working memory, math anxiety, and performance. J. Exp. Psychol. Gen. 130, 224 10.1037/0096-3445.130.2.224 11409101

[B7] Asmundson G. Stein M. Larsen D. Walker J. (1994). Neurocognitive function in panic disorder and social phobia patients. Anxiety 1, 201 9160575

[B8] Baas J. M. P. Milstein J. Donlevy M. Grillon C. (2006). Brainstem correlates of defensive states in humans. Biol. Psychiatry 59, 588–593 10.1016/j.biopsych.2005.09.009 16388780

[B9] Baddeley A. D. Warrington E. K. (1970). Amnesia and the distinction between long- and short-term memory. J. Verbal Learn. Verbal Behav. 9, 176–189

[B10] Bar-Haim Y. Holoshitz Y. Eldar S. Frenkel T. Muller D. Charney D. (2010). Life-threatening danger and suppression of attention bias to threat. Am. J. Psychiatry 167, 694–698 10.1176/appi.ajp.2009.09070956 20395400

[B11] Bar-Haim Y. Lamy D. Pergamin L. Bakermans-Kranenburg M. J. Van I. M. H. (2007). Threat-related attentional bias in anxious and nonanxious individuals: a meta-analytic study. Psychol. Bull. 133, 1–24 10.1037/0033-2909.133.1.1 17201568

[B12] Beddington J. Cooper C. L. Field J. Goswami U. Huppert F. A. Jenkins R. (2008). The mental wealth of nations. Nature 455, 1057–1060 10.1038/4551057a 18948946

[B13] Bishop S. J. (2007). Neurocognitive mechanisms of anxiety: an integrative account. Trends Cogn. Sci. 11, 307–316 10.1016/j.tics.2007.05.008 17553730

[B14] Bishop S. J. (2009). Trait anxiety and impoverished prefrontal control of attention. Nat. Neurosci. 12, 92–98 10.1038/nn.2242 19079249

[B15] Bishop S. J. Jenkins R. Lawrence A. D. (2007). Neural processing of fearful faces: effects of anxiety are gated by perceptual capacity limitations. Cereb. Cortex 17, 1595–1603 10.1093/cercor/bhl070 16956980

[B16] Blair K. Shaywitz J. Smith B. W. Rhodes R. Geraci M. Jones M. (2008). Response to emotional expressions in generalized social phobia and generalized anxiety disorder: evidence for separate disorders. Am. J. Psychiatry 165, 1193 10.1176/appi.ajp.2008.07071060 18483136 PMC2855133

[B17] Blumenthal T. D. Michael E. Dawson A. M. S. Bohmelt A. H. (1999). Short lead interval startle modification, in Startle Modification, ed Blumenthal T. D. (Cambridge: Cambridge University Press), 51–71

[B18] Boldrini M. Del Pace L. Placidi G. P. A. Keilp J. Ellis S. P. Signori S. (2005). Selective cognitive deficits in obsessive-compulsive disorder compared to panic disorder with agoraphobia. Acta Psychiatr. Scand. 111, 150–158 10.1111/j.1600-0447.2004.00247.x 15667435

[B19] Booth R. Sharma D. (2009). Stress reduces attention to irrelevant information: evidence from the Stroop task. Motiv. Emot. 33, 412–418

[B20] Braff D. L. Geyer M. A. Swerdlow N. R. (2001). Human studies of prepulse inhibition of startle: normal subjects, patient groups, and pharmacological studies. Psychopharmacology 156, 234–258 11549226 10.1007/s002130100810

[B21] Braver T. S. (2012). The variable nature of cognitive control: a dual-mechanisms framework. Trends Cogn. Sci. 16, 106–113 10.1016/j.tics.2011.12.010 22245618 PMC3289517

[B22] Browning M. Holmes E. Harmer C. (2010). The modification of attentional bias to emotional information: a review of the techniques, mechanisms, and relevance to emotional disorders. Cogn. Affect. Behav. Neurosci. 10, 8–20 10.3758/CABN.10.1.8 20233952

[B23] Browning M. Holmes E. A. Charles M. Cowen P. J. Harmer C. J. (2012). Using attentional bias modification as a cognitive vaccine against depression. Biol. Psychiatry 72, 572–579 10.1016/j.biopsych.2012.04.014 22579509 PMC3504298

[B24] Bublatzky F. Flaisch T. Stockburger J. Schmälzle R. Schupp H. T. (2010). The interaction of anticipatory anxiety and emotional picture processing: an event-related brain potential study. Psychophysiology 47, 687–696 10.1111/j.1469-8986.2010.00966.x 20136732

[B25] Buckley T. C. Blanchard E. B. Neill W. T. (2000). Information processing and ptsd: a review of the empirical literature. Clin. Psychol. Rev. 20, 1041–1065 10.1016/S0272-7358(99)00030-6 11098399

[B26] Buhle J. Wager T. Smith E. (2010). Using the Stroop task to study emotion regulation, in Self Control in Society, Mind, and Brain, eds Hassin R. Ochsner K. Trope Y. (Oxford: Oxford University Press), 93–113

[B27] Cahill L. Gorski L. Le K. (2003). Enhanced human memory consolidation with post-learning stress: interaction with the degree of arousal at encoding. Learn. Mem. 10, 270–274 10.1101/lm.62403 12888545 PMC202317

[B28] Cahill L. McGaugh J. L. (1998). Mechanisms of emotional arousal and lasting declarative memory. Trends Neurosci. 21, 294–299 10.1016/S0166-2236(97)01214-9 9683321

[B29] Callaway E. (1959). The influence of amobarbital (amylobarbitone) and methamphetamine on the focus of attention. Br. J. Psychiatry 105, 382–392 13665299 10.1192/bjp.105.439.382

[B30] Carver C. S. White T. L. (1994). Behavioral inhibition, behavioral activation, and affective responses to impending reward and punishment: the BIS/BAS Scales. J. Pers. Soc. Psychol. 67, 319

[B31] Chajut E. Algom D. (2003). Selective attention improves under stress: implications for theories of social cognition. J. Pers. Soc. Psychol. 85, 231 10.1037/0022-3514.85.2.231 12916567

[B32] Chechko N. Wehrle R. Erhardt A. Holsboer F. Czisch M. Sãmann P. G. (2009). Unstable prefrontal response to emotional conflict and activation of lower limbic structures and brainstem in remitted panic disorder. PLoS ONE 4:e5537 10.1371/journal.pone.0005537 19462002 PMC2680057

[B33] Chen Y. P. Ehlers A. Clark D. M. Mansell W. (2002). Patients with generalized social phobia direct their attention away from faces. Behav. Res. Ther. 40, 677–687 12051486 10.1016/s0005-7967(01)00086-9

[B34] Chiles W. D. (1958). Effects of shock-induced stress on verbal performance. J. Exp. Psychol. 56, 159 13575713 10.1037/h0042915

[B35] Choi J. Padmala S. Pessoa L. (2012). Impact of state anxiety on the interaction between threat monitoring and cognition. Neuroimage 59, 1912–1923 10.1016/j.neuroimage.2011.08.102 21939773 PMC3230669

[B36] Cisler J. M. Koster E. H. (2010). Mechanisms of attentional biases towards threat in the anxiety disorders: an integrative review. Clin. Psychol. Rev. 30, 203 10.1016/j.cpr.2009.11.003 20005616 PMC2814889

[B37] Clark L. Li R. Wright C. M. Rome F. Fairchild G. Dunn B. D. (2012). Risk-avoidant decision making increased by threat of electric shock. Psychophysiology 49, 1436–1443 10.1111/j.1469-8986.2012.01454.x 22913418

[B38] Clarke P. J. Macleod C. Guastella A. J. (2013). Assessing the role of spatial engagement and disengagement of attention in anxiety-linked attentional bias: a critique of current paradigms and suggestions for future research directions. Anxiety Stress Coping 26, 1–19 10.1080/10615806.2011.638054 22136158

[B39] Cohen J. D. Perlstein W. M. Braver T. S. Nystrom L. E. Noll D. C. Jonides J. (1997). Temporal dynamics of brain activation during a working memory task. Nature 386, 604–608 10.1038/386604a0 9121583

[B40] Cohen L. J. Hollander E. Decaria C. M. Stein D. J. (1996). Specificity of neuropsychological impairment in obsessive-compulsive disorder: a comparison with social phobic and normal control subjects. J. Neuropsychiatry Clin. Neurosci. 8, 82–85 8845706 10.1176/jnp.8.1.82

[B41] Cools R. Calder A. Lawrence A. Clark L. Bullmore E. Robbins T. (2005). Individual differences in threat sensitivity predict serotonergic modulation of amygdala response to fearful faces. Psychopharmacology 180, 670–679 10.1007/s00213-005-2215-5 15772862

[B42] Cornwell B. R. Alvarez R. P. Lissek S. Kaplan R. Ernst M. Grillon C. (2011). Anxiety overrides the blocking effects of high perceptual load on amygdala reactivity to threat-related distractors. Neuropsychologia 49, 1363–1368 10.1016/j.neuropsychologia.2011.02.049 21376745 PMC3085886

[B43] Cornwell B. R. Arkin N. Overstreet C. Carver F. W. Grillon C. (2012a). Distinct contributions of human hippocampal theta to spatial cognition and anxiety. Hippocampus 22, 1848–1859 10.1002/hipo.22019 22467298 PMC3390451

[B44] Cornwell B. R. Mueller S. C. Kaplan R. Grillon C. Ernst M. (2012b). Anxiety, a benefit and detriment to cognition: behavioral and magnetoencephalographic evidence from a mixed-saccade task. Brain Cogn. 78, 257–267 10.1016/j.bandc.2012.01.002 22289426 PMC3448553

[B45] Cornwell B. R. Baas J. M. P. Johnson L. Holroyd T. Carver F. W. Lissek S. (2007). Neural responses to auditory stimulus deviance under threat of electric shock revealed by spatially-filtered magnetoencephalography. Neuroimage 37, 282–289 10.1016/j.neuroimage.2007.04.055 17566766 PMC2717627

[B46] Cornwell B. R. Echiverri A. M. Covington M. F. Grillon C. (2008). Modality-specific attention under imminent but not remote threat of shock: evidence from differential prepulse inhibition of startle. Psychol. Sci. 19, 615–622 10.1111/j.1467-9280.2008.02131.x 18578853 PMC2680742

[B47] Corr P. J. Tynan A. Kumari V. (2002). Personality correlates of prepulse inhibition of the startle reflex at three lead intervals. J. Psychophysiol. 16, 82–91

[B48] Darke S. (1988). Anxiety and working memory capacity. Cogn. Emot. 2, 145–154

[B49] Davis M. (1998). Are different parts of the extended amygdala involved in fear versus anxiety? Biol. Psychiatry 44, 1239–1247 9861467 10.1016/s0006-3223(98)00288-1

[B50] Davis M. Walker D. L. Miles L. Grillon C. (2010). Phasic vs sustained fear in rats and humans: role of the extended amygdala in fear vs anxiety. Neuropsychopharmacology 35, 105–135 10.1038/npp.2009.109 19693004 PMC2795099

[B51] Dawson G. R. Goodwin G. (2005). Experimental medicine in psychiatry. J. Psychopharmacol. 19, 565–566 10.1177/0269881105059935 16272178

[B52] De Pascalis V. Cozzuto G. Russo E. (2013). Effects of personality trait emotionality on acoustic startle response and prepulse inhibition including N100 and P200 event-related potential. Clin. Neurophysiol. 124, 292–305 10.1016/j.clinph.2012.07.018 22938794

[B53] De Ruiter C. Brosschot J. F. (1994). The emotional Stroop interference effect in anxiety: attentional bias or cognitive avoidance? Behav. Res. Ther. 32, 315–319 10.1016/0005-7967(94)90128-7 8192630

[B54] de Visser L. van der Knaap L. J. van de Loo A. J. aE. van der Weerd C. M. M. Ohl F. van den Bos R. (2010). Trait anxiety affects decision-making differently in healthy men and women: towards gender-specific endophenotypes of anxiety. Neuropsychologia 48, 1598–1606 10.1016/j.neuropsychologia.2010.01.027 20138896

[B55] Derakshan N. Eysenck M. W. (1998). Working memory capacity in high trait-anxious and repressor groups. Cogn. Emot. 12, 697–713

[B56] Derryberry D. Reed M. A. (2002). Anxiety-related attentional biases and their regulation by attentional control. J. Abnorm. Psychol. 111, 225 10.1037/0021-843X.111.2.225 12003445

[B57] Duley A. R. Hillman C. H. Coombes S. Janelle C. M. (2007). Sensorimotor gating and anxiety: prepulse inhibition following acute exercise. Int. J. Psychophysiol. 64, 157–164 10.1016/j.ijpsycho.2007.01.006 17350126

[B58] Duncko R. Cornwell B. Cui L. Merikangas K. R. Grillon C. (2007). Acute exposure to stress improves performance in trace eyeblink conditioning and spatial learning tasks in healthy men. Learn. Mem. 14, 329–335 10.1101/lm.483807 17522023 PMC1876756

[B59] Duncko R. Johnson L. Merikangas K. Grillon C. (2009). Working memory performance after acute exposure to the cold pressor stress in healthy volunteers. Neurobiol. Learn. Mem. 91, 377–381 19340949 10.1016/j.nlm.2009.01.006 PMC2696884

[B60] Eason R. G. Harter M. R. White C. T. (1969). Effects of attention and arousal on visually evoked cortical potentials and reaction time in man. Physiol. Behav. 4, 283–289

[B61] Easterbrook J. A. (1959). The effect of emotion on cue utilization and the organization of behavior. Psychol. Rev. 66, 183–201 13658305 10.1037/h0047707

[B62] Edwards M. Burt J. Lipp O. (2010). Selective attention for masked and unmasked emotionally toned stimuli: effects of trait anxiety, state anxiety, and test order. Br. J. Psychol. 101, 325–343 10.1348/000712609X466559 19709474

[B63] Edwards M. S. Burt J. S. Lipp O. V. (2006). Selective processing of masked and unmasked verbal threat material in anxiety: Influence of an immediate acute stressor. Cogn. Emot. 20, 812–835

[B64] Ekstrom A. D. Kahana M. J. Caplan J. B. Fields T. A. Isham E. A. Newman E. L. (2003). Cellular networks underlying human spatial navigation. Nature 425, 184–188 10.1038/nature01964 12968182

[B65] Elliott R. Baker S. C. Rogers R. D. O'Leary D. A. Paykel E. S. Frith C. D. (1997). Prefrontal dysfunction in depressed patients performing a complex planning task: a study using positron emission tomography. Psychol. Med. 27, 931–942 9234470 10.1017/s0033291797005187

[B66] Etkin A. (2010). Functional neuroanatomy of anxiety: a neural circuit perspective. Curr. Top. Behav. Neurosci. 2, 251–277 21309113 10.1007/7854_2009_5

[B67] Etkin A. Egner T. Kalisch R. (2011). Emotional processing in anterior cingulate and medial prefrontal cortex. Trends Cogn. Sci. 15, 85–93 10.1016/j.tics.2010.11.004 21167765 PMC3035157

[B68] Etkin A. Egner T. Peraza D. M. Kandel E. R. Hirsch J. (2006). Resolving emotional conflict: a role for the rostral anterior cingulate cortex in modulating activity in the amygdala. Neuron 51, 871–882 10.1016/j.neuron.2006.07.029 16982430

[B69] Etkin A. Prater K. E. Hoeft F. Menon V. Schatzberg A. F. (2010). Failure of anterior cingulate activation and connectivity with the amygdala during implicit regulation of emotional processing in generalized anxiety disorder. Am. J. Psychiatry 167, 545–554 10.1176/appi.ajp.2009.09070931 20123913 PMC4367202

[B70] Etkin A. Schatzberg A. (2012). Common abnormalities and disorder-specific compensation during implicit regulation of emotional processing in generalized anxiety and major depressive disorders. Am. J. Psychiatry 168, 968–978 10.1176/appi.ajp.2011.10091290 21632648

[B71] Etkin A. Wager T. D. (2007). Functional neuroimaging of anxiety: a meta-analysis of emotional processing in PTSD, social anxiety disorder, and specific phobia. Am. J. Psychiatry 164, 1476–1488 10.1176/appi.ajp.2007.07030504 17898336 PMC3318959

[B72] Eysenck M. Payne S. Derakshan N. (2005). Trait anxiety, visuospatial processing, and working memory. Cogn. Emot. 19, 1214–1228 10.1080/10615800701847823 18686056

[B73] Eysenck M. W. Calvo M. G. (1992). Anxiety and performance: the processing efficiency theory. Cogn. Emot. 6, 409–434

[B74] Eysenck M. W. Derakshan N. Santos R. Calvo M. G. (2007). Anxiety and cognitive performance: attentional control theory. Emotion 7, 336 10.1037/1528-3542.7.2.336 17516812

[B75] Fales C. Barch D. Burgess G. Schaefer A. Mennin D. Gray J. (2008). Anxiety and cognitive efficiency: differential modulation of transient and sustained neural activity during a working memory task. Cogn. Affect. Behav. Neurosci. 8, 239–253 18814461 10.3758/cabn.8.3.239

[B76] Fineberg N. A. Potenza M. N. Chamberlain S. R. Berlin H. A. Menzies L. Bechara A. (2009). Probing compulsive and impulsive behaviors, from animal models to endophenotypes: a narrative review. Neuropsychopharmacology 35, 591–604 10.1038/npp.2009.185 19940844 PMC3055606

[B77] Firetto A. C. Davey H. (1971). Subjectively reported anxiety as a discriminator of digit span performance. Psychol. Rep. 28, 98 5549464 10.2466/pr0.1971.28.1.98

[B78] Franklin J. C. Bowker K. B. Blumenthal T. D. (2009). Anxiety and prepulse inhibition of acoustic startle in a normative sample: the importance of signal-to-noise ratio. Pers. Individ. Dif. 46, 369–373

[B79] Friedman B. H. Thayer J. F. Borkovec T. D. (2000). Explicit memory bias for threat words in generalized anxiety disorder. Behav. Ther. 31, 745–756

[B80] Friedman N. P. Miyake A. (2004). The relations among inhibition and interference control functions: a latent-variable analysis. J. Exp. Psychol. Gen. 133, 101 10.1037/0096-3445.133.1.101 14979754

[B81] Garner M. Mogg K. Bradley B. P. (2006). Orienting and maintenance of gaze to facial expressions in social anxiety. J. Abnorm. Psychol. 115, 760–770 10.1037/0021-843X.115.4.760 17100533

[B82] Ge Y. Wu J. Sun X. Zhang K. (2011). Enhanced mismatch negativity in adolescents with posttraumatic stress disorder (PTSD). Int. J. Psychophysiol. 79, 231–235 10.1016/j.ijpsycho.2010.10.012 21036193

[B83] Gladsjo J. A. Rapaport M. H. McKinney R. Lucas J. A. Rabin A. Oliver T. (1998). A neuropsychological study of panic disorder: negative findings. J. Affect. Disord. 49, 123–131 9609676 10.1016/s0165-0327(98)00006-8

[B84] Grillon C. (2002). Startle reactivity and anxiety disorders: aversive conditioning, context, and neurobiology. Biol. Psychiatry 52, 958–975 12437937 10.1016/s0006-3223(02)01665-7

[B85] Grillon C. (2008). Models and mechanisms of anxiety: evidence from startle studies. Psychopharmacology 199, 421–437 10.1007/s00213-007-1019-1 18058089 PMC2711770

[B86] Grillon C. Ameli R. Woods S. W. Merikangas K. Davis M. (1991). Fear-potentiated startle in humans: effects of anticipatory anxiety on the acoustic blink reflex. Psychophysiology 28, 588–595 1758934 10.1111/j.1469-8986.1991.tb01999.x

[B87] Grillon C. Charney D. R. (2011). In the face of fear: anxiety sensitizes defensive responses to fearful faces. Psychophysiology 48, 1745–1752 10.1111/j.1469-8986.2011.01268.x 21824155 PMC3212615

[B88] Grillon C. Davis M. (2007). Effects of stress and shock anticipation on prepulse inhibition of the startle reflex. Psychophysiology 34, 511–517 9299905 10.1111/j.1469-8986.1997.tb01737.x

[B89] Grillon C. Dierker L. Merikangas K. R. (1997). Startle modulation in children at risk for anxiety disorders and/or alcoholism. J. Am. Acad. Child Adolesc. Psychiatry 36, 925–932 10.1097/00004583-199707000-00014 9204670

[B90] Grillon C. Morgan C. A. Southwick S. M. Davis M. Charney D. S. (1996). Baseline startle amplitude and prepulse inhibition in Vietnam veterans with posttraumatic stress disorder. Psychiatry Res. 64, 169–178 10.1016/S0165-1781(96)02942-3 8944395

[B91] Haas B. W. Omura K. Constable R. T. Canli T. (2006). Interference produced by emotional conflict associated with anterior cingulate activation. Cogn. Affect. Behav. Neurosci. 6, 152–156 17007235 10.3758/cabn.6.2.152

[B92] Hajcak G. Foti D. (2008). Errors are aversive defensive motivation and the error-related negativity. Psychol. Sci. 19, 103–108 10.1111/j.1467-9280.2008.02053.x 18271855

[B93] Hakamata Y. Lissek S. Bar-Haim Y. Britton J. C. Fox N. A. Leibenluft E. (2010). Attention bias modification treatment: a meta-analysis toward the establishment of novel treatment for anxiety. Biol. Psychiatry 68, 982–990 10.1016/j.biopsych.2010.07.021 20887977 PMC3296778

[B94] Hansen A. L. Johnsen B. H. Thayer J. F. (2009). Relationship between heart rate variability and cognitive function during threat of shock. Anxiety Stress Coping 22, 77–89 10.1080/10615800802272251 18781457

[B95] Hansenne M. Pinto E. Scantamburlo G. Renard B. Reggers J. Fuchs S. (2003). Harm avoidance is related to mismatch negativity (MMN) amplitude in healthy subjects. Pers. Individ. Dif. 34, 1039–1048

[B96] Harmer C. J. Cowen P. J. Goodwin G. M. (2011). Efficacy markers in depression. J. Psychopharmacol. 25, 1148–1158 10.1177/0269881110367722 20530590

[B97] Hartley C. A. Phelps E. A. (2012). Anxiety and decision-making. Biol. Psychiatry 72, 113–118 10.1016/j.biopsych.2011.12.027 22325982 PMC3864559

[B98] Haxby J. V. Hoffman E. A. Gobbini M. I. (2000). The distributed human neural system for face perception. Trends Cogn. Sci. 4, 223–233 10.1016/S1364-6613(00)01482-0 10827445

[B99] Helfinstein S. White L. Bar Haim Y. Fox N. (2008). Affective primes suppress attention bias to threat in socially anxious individuals. Behav. Res. Ther. 46, 799–810 10.1016/j.brat.2008.03.011 18472088 PMC2527506

[B100] Hockey G. M. Gaillard A. W. K. Coles M. G. H. (1986). Energetics and human information processing, in Proceedings of the NATO Advanced Research Workshop, (Les Arcs: Springer).

[B101] Hodges W. F. Durham R. L. (1972). Anxiety, ability, and digit span performance. J. Pers. Soc. Psychol. 24, 401–406 4655548 10.1037/h0033732

[B102] Hoenig K. Hochrein A. Quednow B. B. Maier W. Wagner M. (2005). Impaired prepulse inhibition of acoustic startle in obsessive-compulsive disorder. Biol. Psychiatry 57, 1153–1158 10.1016/j.biopsych.2005.01.040 15866555

[B103] Hu K. Bauer A. Padmala S. Pessoa L. (2012). Threat of bodily harm has opposing effects on cognition. Emotion 12, 28–32 10.1037/a0024345 21707143 PMC3207017

[B104] Ikeda M. Iwanaga M. Seiwa H. (1996). Test anxiety and working memory system. Percept. Mot. Skills 82, 1223–1231 8823887 10.2466/pms.1996.82.3c.1223

[B105] Ishizuka K. Hillier A. Beversdorf D. Q. (2007). Effect of the cold pressor test on memory and cognitive flexibility. Neurocase 13, 154–157 10.1080/13554790701441403 17786773

[B106] Jarcho J. M. Fox N. A. Pine D. S. Etkin A. Leibenluft E. Shechner T. (2013). The neural correlates of emotion-based cognitive control in adults with early childhood behavioral inhibition. Biol. Psychol. 92, 306–314 10.1016/j.biopsycho.2012.09.008 23046903 PMC3558536

[B107] Jonides J. Lewis R. L. Nee D. E. Lustig C. A. Berman M. G. Moore K. S. (2008). The mind and brain of short-term memory. Annu. Rev. Psychol. 59, 193–224 10.1146/annurev.psych.59.103006.093615 17854286 PMC3971378

[B108] Kalin N. Shelton S. (1989). Defensive behaviors in infant rhesus monkeys: environmental cues and neurochemical regulation. Science 243, 1718–1721 10.1126/science.2564702 2564702

[B109] Kalisch R. Wiech K. Critchley H. D. Dolan R. J. (2006). Levels of appraisal: a medial prefrontal role in high-level appraisal of emotional material. Neuroimage 30, 1458–1466 10.1016/j.neuroimage.2005.11.011 16388969

[B110] Keinan G. (1987). Decision making under stress: scanning of alternatives under controllable and uncontrollable threats. J. Pers. Soc. Psychol. 52, 639–644 10.1037/0022-3514.52.3.639 3572731

[B111] Kenemans J. L. Wieleman J. S. Zeegers M. Verbaten M. N. (1999). Caffeine and Stroop interference. Pharmacol. Biochem. Behav. 63, 589–598 10.1016/S0091-3057(99)00022-2 10462187

[B112] Kessler R. C. Avenevoli S. McLaughlin K. A. Green J. G. Lakoma M. D. Petukhova M. (2012). Lifetime co-morbidity of DSM-IV disorders in the US National Comorbidity Survey Replication Adolescent Supplement (NCS-A). Psychol. Med. 42, 1997–2010 10.1017/S0033291712000025 22273480 PMC3448706

[B113] Kizilbash A. H. Vanderploeg R. D. Curtiss G. (2002). The effects of depression and anxiety on memory performance. Arch. Clin. Neuropsychol. 17, 57–67 10.1016/S0887-6177(00)00101-3 14589753

[B114] Knott V. J. Bakish D. Barkley J. (1994). Brainstem evoked potentials in panic disorder. J. Psychiatry Neurosci. 19, 301 7918353 PMC1188612

[B115] Kola I. Landis J. (2004). Can the pharmaceutical industry reduce attrition rates? Nat. Rev. Drug Discov. 3, 711–716 10.1038/nrd1470 15286737

[B116] Krug M. K. Carter C. S. (2012). Proactive and reactive control during emotional interference and its relationship to trait anxiety. Brain Res. 1481, 13–36 10.1016/j.brainres.2012.08.045 22960116 PMC3541031

[B117] Kuhajda M. Thorn B. Klinger M. (1998). The effect of pain on memory for affective words. Ann. Behav. Med. 20, 31–35 9755349 10.1007/BF02893806

[B118] Kuhlmann S. Piel M. Wolf O. T. (2005). Impaired memory retrieval after psychosocial stress in healthy young men. J. Neurosci. 25, 2977–2982 10.1523/JNEUROSCI.5139-04.2005 15772357 PMC6725125

[B119] Lagarde G. V. Doyon J. Brunet A. (2010). Memory and executive dysfunctions associated with acute posttraumatic stress disorder. Psychiatry Res. 177, 144–149 10.1016/j.psychres.2009.02.002 20381880

[B120] Lapointe M.-L. B. Blanchette I. Duclos M. Langlois F. Provencher M. D. Tremblay S. (2013). Attentional bias, distractibility and short-term memory in anxiety. Anxiety Stress Coping 26, 293–313 10.1080/10615806.2012.687722 22762442

[B121] Laretzaki G. Plainis S. Argyropoulos S. Pallikaris I. Bitsios P. (2010). Threat and anxiety affect visual contrast perception. J. Psychopharmacol. 24, 667–675 10.1177/0269881108098823 19010976

[B122] Lavie N. Hirst A. De Fockert J. Viding E. (2004). Load theory of selective attention and cognitive control. J. Exp. Psychol. Gen. 133, 339–354 10.1037/0096-3445.133.3.339 15355143

[B123] Lavric A. Rippon G. Gray J. R. (2003). Threat-evoked anxiety disrupts spatial working memory performance: an attentional account. Cogn. Ther. Res. 27, 489–504

[B124] Ledoux J. (1998). The Emotional Brain: The Mysterious Underpinnings of Emotional Life. New York, NY: Simon and Schuster

[B125] Lighthall N. R. Mather M. Gorlick M. A. (2009). Acute stress increases sex differences in risk seeking in the balloon analogue risk task. PLoS ONE 4:e6002 10.1371/journal.pone.0006002 19568417 PMC2698212

[B126] Lighthall N. R. Sakaki M. Vasunilashorn S. Nga L. Somayajula S. Chen E. Y. (2012). Gender differences in reward-related decision processing under stress. Soc. Cogn. Affect. Neurosci. 7, 476–484 10.1093/scan/nsr026 21609968 PMC3324572

[B127] Lindström B. R. Bohlin G. (2012). Threat-relevance impairs executive functions: negative impact on working memory and response inhibition. Emotion 12, 384–393 10.1037/a0027305 22468619

[B128] Linnman C. Zeidan M. A. Furtak S. C. Pitman R. K. Quirk G. J. Milad M. R. (2012). Resting amygdala and medial prefrontal metabolism predicts functional activation of the fear extinction circuit. Am. J. Psychiatry 169, 415–423 10.1176/appi.ajp.2011.10121780 22318762 PMC4080711

[B129] Lipschitz D. S. Mayes L. M. Rasmusson A. M. Anyan W. Billingslea E. Gueorguieva R. (2005). Baseline and modulated acoustic startle responses in adolescent girls with posttraumatic stress disorder. J. Am. Acad. Child Adolesc. Psychiatry 44, 807 10.1097/01.chi.0000166379.60769.b6 16034283

[B130] Lissek S. Rabin S. Heller R. E. Lukenbaugh D. Geraci M. Pine D. S. (2009). Overgeneralization of conditioned fear as a pathogenic marker of panic disorder. Am. J. Psychiatry 167, 47–55 10.1176/appi.ajp.2009.09030410 19917595 PMC2806514

[B131] Litz B. T. Weathers F. W. Monaco V. Herman D. S. Wulfsohn M. Marx B. (1996). Attention, arousal, and memory in posttraumatic stress disorder. J. Trauma. Stress 9, 497–519 8827652 10.1007/BF02103661

[B132] Lucas J. A. Telch M. J. Bigler E. D. (1991). Memory functioning in panic disorder: a neuropsychological perspective. J. Anxiety Disord. 5, 1–20

[B133] Ludewig S. Ludewig K. Geyer M. Hell D. Vollenweider F. (2002). Prepulse inhibition deficits in patients with panic disorder. Depress. Anxiety 15, 55–60 10.1002/da.10026 11891993

[B134] Macleod C. Donnellan A. M. (1993). Individual differences in anxiety and the restriction of working memory capacity. Pers. Individ. Dif. 15, 163–173

[B135] Macleod C. Mathews A. (1988). Anxiety and the allocation of attention to threat. Q. J. Exp. Psychol. A 40, 653–670 3212208 10.1080/14640748808402292

[B136] Macleod C. Mathews A. (2012). Cognitive bias modification approaches to anxiety. Annu. Rev. Clin. Psychol. 8, 189–217 10.1146/annurev-clinpsy-032511-143052 22035241

[B137] Macleod C. Mathews A. Tata P. (1986). Attentional bias in emotional disorders. J. Abnorm. Psychol. 95, 15–20 10.1037/0021-843X.95.1.15 3700842

[B138] Maier S. Szalkowski A. Kamphausen S. Perlov E. Feige B. Blechert J. (2012). Clarifying the role of the rostral dmPFC/dACC in fear/anxiety: learning, appraisal or expression? PLoS ONE 7:e50120 10.1371/journal.pone.0050120 23189183 PMC3506550

[B139] Maisonnette S. S. Kawasaki M. C. Coimbra N. C. Brandao M. L. (1996). Effects of lesions of amygdaloid nuclei and substantia nigra on aversive responses induced by electrical stimulation of the inferior colliculus. Brain Res. Bull. 40, 93–98 10.1016/0361-9230(95)02136-1 8724425

[B140] Markham R. Darke S. (1991). The effects of anxiety on verbal and spatial task performance. Aust. J. Psychol. 43, 107–111

[B141] Mather M. Gorlick M. A. Lighthall N. R. (2009). To brake or accelerate when the light turns yellow?: stress reduces older adults' risk taking in a driving game. Psychol. Sci. 20, 174–176 10.1111/j.1467-9280.2009.02275.x 19175527 PMC2704238

[B142] Mathews A. Mackintosh B. A. (1998). A cognitive model of selective processing in anxiety. Cogn. Ther. Res. 22, 539–560

[B143] Mathews A. Mogg K. May J. Eysenck M. (1989). Implicit and explicit memory bias in anxiety. J. Abnorm. Psychol. 98, 236 10.1037/0021-843X.98.3.236 2768658

[B144] Mathews A. Sebastian S. (1993). Suppression of emotional Stroop effects by fear-arousal. Cogn. Emot. 7, 517–530

[B145] McNally R. J. Foa E. B. Donnell C. D. (1989). Memory bias for anxiety information in patients with panic disorder. Cogn. Emot. 3, 27–44

[B146a] Mcvay J. C. Kane M. J. (2010). Does mind wandering reflect executive function or executive failure? Comment on Smallwood and Schooler (2006) and Watkins (2008). Psychol. Bull. 136, 188–197 10.1037/a0018298 20192557 PMC2850105

[B146] Menning H. Renz A. Seifert J. Maercker A. (2008). Reduced mismatch negativity in posttraumatic stress disorder: a compensatory mechanism for chronic hyperarousal? Int. J. Psychophysiol. 68, 27–34 10.1016/j.ijpsycho.2007.12.003 18262297

[B147] Miller M. Patrick C. (2000). Trait differences in affective and attentional responding to threat revealed by emotional stroop interference and startle reflex modulation. Behav. Ther. 31, 757–776

[B148] Mineka S. Watson D. Clark L. A. (1998). Comorbidity of anxiety and unipolar mood disorders. Annu. Rev. Psychol. 49, 377–412 10.1146/annurev.psych.49.1.377 9496627

[B149] Miu A. C. Heilman R. M. Houser D. (2008). Anxiety impairs decision-making: Psychophysiological evidence from an Iowa Gambling Task. Biol. Psychol. 77, 353–358 10.1016/j.biopsycho.2007.11.010 18191013

[B150] Mogg K. Bradley B. (2005). Attentional bias in generalized anxiety disorder versus depressive disorder. Cogn. Ther. Res. 29, 29–45 10.1207/S15374424JCCP3201_02 12573928

[B151] Mogg K. Mathews A. Weinman J. (1987). Memory bias in clinical anxiety. J. Abnorm. Psychol. 96, 94 10.1037/0021-843X.96.2.94 3584672

[B152] Monk C. Nelson E. McClure E. Mogg K. Bradley B. Leibenluft E. (2006). Ventrolateral prefrontal cortex activation and attentional bias in response to angry faces in adolescents with generalized anxiety disorder. Am. J. Psychiatry 163, 1091–1097 10.1176/appi.ajp.163.6.1091 16741211

[B153] Morgan C. A. III Grillon C. (1999). Abnormal mismatch negativity in women with sexual assault-related posttraumatic stress disorder. Biol. Psychiatry 45, 827–832 10202569 10.1016/s0006-3223(98)00194-2

[B154] Morris J. S. Ohman A. Dolan R. J. (1999). A subcortical pathway to the right amygdala mediating ‘unseen’ fear. Proc. Natl. Acad. Sci. U.S.A. 96, 1680–1685 10.1073/pnas.96.4.1680 9990084 PMC15559

[B155] Mueller E. M. Nguyen J. Ray W. J. Borkovec T. D. (2010). Future-oriented decision-making in Generalized Anxiety Disorder is evident across different versions of the Iowa Gambling Task. J. Behav. Ther. Exp. Psychiatry 41, 165–171 10.1016/j.jbtep.2009.12.002 20060098

[B156] Mueller S. C. Temple V. Cornwell B. Grillon C. Pine D. S. Ernst M. (2009). Impaired spatial navigation in pediatric anxiety. J. Child Psychol. Psychiatry 50, 1227–1234 10.1111/j.1469-7610.2009.02112.x 19594834 PMC2788776

[B157] Murphy F. Smith K. Cowen P. Robbins T. Sahakian B. (2002). The effects of tryptophan depletion on cognitive and affective processing in healthy volunteers. Psychopharmacology 163, 42–53 10.1007/s00213-002-1128-9 12185399

[B158] Murphy F. C. Sahakian B. J. Rubinsztein J. S. Michael A. Rogers R. D. Robbins T. W. (1999). Emotional bias and inhibitory control processes in mania and depression. Psychol. Med. 29, 1307–1321 10616937 10.1017/s0033291799001233

[B159] Murphy R. E. (1959). Effects of threat of shock, distraction, and task design on performance. J. Exp. Psychol. 58, 134–141 14425409 10.1037/h0044233

[B160] Norton G. R. Schaefer E. Cox B. J. Dorward J. Wozney K. (1988). Selective memory effects in nonclinical panickers. J. Anxiety Disord. 2, 169–177

[B161] Nugent K. Mineka S. (1994). The effect of high and low trait anxiety on implicit and explicit memory tasks. Cogn. Emot. 8, 147–163 8135724

[B162] Oei N. Everaerd W. Elzinga B. Van Well S. Bermond B. (2006). Psychosocial stress impairs working memory at high loads: an association with cortisol levels and memory retrieval. Stress 9, 133–141 10.1080/10253890600965773 17035163

[B163] O'Malley J. J. Gallas J. (1977). Noise and attention span. Percept. Mot. Skills 44, 919–922 876803 10.2466/pms.1977.44.3.919

[B164] O'Malley J. J. Poplawsky A. (1971). Noise-induced arousal and breadth of attention. Percept. Mot. Skills 33, 887–890 5127209 10.2466/pms.1971.33.3.887

[B165] Pallak M. S. Pittman T. S. Heller J. F. Munson P. (1975). The effect of arousal on Stroop color-word task performance. Bull. Psychon. Soc. 8, 248–250

[B166] Paunovic N. Lundh L. G. Öst L. G. (2002). Attentional and memory bias for emotional information in crime victims with acute posttraumatic stress disorder (PTSD). J. Anxiety Disord. 16, 675–692 12405525 10.1016/s0887-6185(02)00136-6

[B167] Pessoa L. (2005). To what extent are emotional visual stimuli processed without attention and awareness? Curr. Opin. Neurobiol. 15, 188–196 10.1016/j.conb.2005.03.002 15831401

[B168] Pessoa L. (2009). How do emotion and motivation direct executive control? Trends Cogn. Sci. 13, 160–166 10.1016/j.tics.2009.01.006 19285913 PMC2773442

[B169] Pessoa L. Gutierrez E. Bandettini P. A. Ungerleider L. G. (2002). Neural correlates of visual working memory: fMRI amplitude predicts task performance. Neuron 35, 975–987 10.1016/S0896-6273(02)00817-6 12372290

[B170] Pessoa L. Padmala S. Morland T. (2005). Fate of unattended fearful faces in the amygdala is determined by both attentional resources and cognitive modulation. Neuroimage 28, 249–255 10.1016/j.neuroimage.2005.05.048 15993624 PMC2427145

[B171] Petry N. M. Stinson F. S. Grant B. F. (2005). Comorbidity of DSM-IV pathological gambling and other psychiatric disorders: results from the national epidemiologic survey on alcohol and related conditions. J. Clin. Psychiatry 66, 564–574 15889941 10.4088/jcp.v66n0504

[B172] Phillips M. L. Drevets W. C. Rauch S. L. Lane R. (2003). Neurobiology of emotion perception I: the neural basis of normal emotion perception. Biol. Psychiatry 54, 504–514 10.1016/S0006-3223(03)00168-9 12946879

[B173] Porcelli A. J. Cruz D. Wenberg K. Patterson M. D. Biswal B. B. Rypma B. (2008). The effects of acute stress on human prefrontal working memory systems. Physiol. Behav. 95, 282–289 10.1016/j.physbeh.2008.04.027 18692209

[B174] Porcelli A. J. Delgado M. R. (2009). Acute stress modulates risk taking in financial decision making. Psychol. Sci. 20, 278–283 10.1111/j.1467-9280.2009.02288.x 19207694 PMC4882097

[B175] Postle B. R. (2006). Working memory as an emergent property of the mind and brain. Neuroscience 139, 23–38 10.1016/j.neuroscience.2005.06.005 16324795 PMC1428794

[B176] Preston S. D. Buchanan T. W. Stansfield R. B. Bechara A. (2007). Effects of anticipatory stress on decision making in a gambling task. Behav. Neurosci. 121, 257–263 10.1037/0735-7044.121.2.257 17469915

[B177] Pyke S. Agnew N. M. (1963). Digit span performance as a function of noxious stimulation. J. Consult. Psychol. 27, 281 13972641 10.1037/h0041884

[B178] Qin S. Hermans E. J. Van Marle H. J. F. Luo J. Fernández G. (2009). Acute psychological stress reduces working memory-related activity in the dorsolateral prefrontal cortex. Biol. Psychiatry 66, 25–32 10.1016/j.biopsych.2009.03.006 19403118

[B179] Reeb-Sutherland B. C. Vanderwert R. E. Degnan K. A. Marshall P. J. Pérez-Edgar K. Chronis-Tuscano A. (2009). Attention to novelty in behaviorally inhibited adolescents moderates risk for anxiety. J. Child Psychol. Psychiatry 50, 1365–1372 10.1111/j.1469-7610.2009.02170.x 19788588 PMC2851743

[B180] Reidy J. Richards A. (1997). Anxiety and memory: a recall bias for threatening words in high anxiety. Behav. Res. Ther. 35, 531–542 10.1016/S0005-7967(97)00001-6 9159977

[B181] Richards A. French C. C. Keogh E. Carter C. (2000). Test-Anxiety, inferential reasoning and working memory load. Anxiety Stress Coping 13, 87–109

[B182] Richardson R. (2000). Shock sensitization of startle: learned or unlearned fear? Behav. Brain Res. 110, 109–117 10.1016/S0166-4328(99)00189-8 10802308

[B183] Robinson O. J. Charney D. R. Overstreet C. Vytal K. Grillon C. (2012a). The adaptive threat bias in anxiety: amygdala–dorsomedial prefrontal cortex coupling and aversive amplification. Neuroimage 60, 523–529 10.1016/j.neuroimage.2011.11.096 22178453 PMC3288162

[B184] Robinson O. J. Overstreet C. Letkiewicz A. Grillon C. (2012b). Depressed mood enhances anxiety to unpredictable threat. Psychol. Med. 42, 1397–1407 10.1017/S0033291711002583 22088577 PMC3288206

[B185] Robinson O. J. Krimpsky M. Grillon C. (2013a). The impact of anxiety on response inhibition. Front. Hum. Neurosci. 7:69 10.3389/fnhum.2013.00069 23471118 PMC3590569

[B186] Robinson O. J. Overstreet C. Charney D. S. Vytal K. Grillon C. (2013b). Stress increases aversive prediction-error signal in the ventral striatum. Proc. Natl. Acad. Sci. U.S.A. 110, 4129–4133 10.1073/pnas.1213923110 23401511 PMC3593853

[B187] Robinson O. J. Letkiewicz A. M. Overstreet C. Ernst M. Grillon C. (2011). The effect of induced anxiety on cognition: threat of shock enhances aversive processing in healthy individuals. Cogn. Affect. Behav. Neurosci. 11, 217–227 10.3758/s13415-011-0030-5 21484411 PMC3169349

[B188] Robinson O. J. Sahakian B. J. (2009). A double dissociation in the roles of serotonin and mood in healthy subjects. Biol. Psychiatry 65, 89–92 10.1016/j.biopsych.2008.10.001 18996509 PMC2602857

[B189] Rodgers R. (1997). Animal models of ‘anxiety’: where next? Behav. Pharmacol. 8, 477–496 9832964 10.1097/00008877-199711000-00003

[B190] Roozendaal B. (2002). Stress and memory: opposing effects of glucocorticoids on memory consolidation and memory retrieval. Neurobiol. Learn. Mem. 78, 578–595 10.1006/nlme.2002.4080 12559837

[B191] Roozendaal B. McEwen B. S. Chattarji S. (2009). Stress, memory and the amygdala. Nat. Rev. Neurosci. 10, 423–433 10.1038/nrn2651 19469026

[B192] Roozendaal B. Okuda S. van der Zee E. A. McGaugh J. L. (2006). Glucocorticoid enhancement of memory requires arousal-induced noradrenergic activation in the basolateral amygdala. Proc. Natl. Acad. Sci. U.S.A. 103, 6741–6746 10.1073/pnas.0601874103 16611726 PMC1458951

[B193] Roy A. K. Vasa R. A. Bruck M. Mogg K. Bradley B. P. Sweeney M. (2008). Attention bias toward threat in pediatric anxiety disorders. J. Am. Acad. Child Adolesc. Psychiatry 47, 1189 10.1097/CHI.0b013e3181825ace 18698266 PMC2783849

[B194] Salemink E. Van Den Hout M. Kindt M. (2009). Effects of positive interpretive bias modification in highly anxious individuals. J. Anxiety Disord. 23, 676–683 10.1016/j.janxdis.2009.02.006 19272750

[B195] Sauro M. D. Jorgensen R. S. Teal Pedlow C. (2003). Stress, glucocorticoids, and memory: a meta-analytic review. Stress 6, 235–245 10.1080/10253890310001616482 14660056

[B196] Schmitz A. Grillon C. (2012). Assessing fear and anxiety in humans using the threat of predictable and unpredictable aversive events (the NPU-threat test). Nat. Protoc. 7, 527–532 10.1038/nprot.2012.001 22362158 PMC3446242

[B197] Schoofs D. Preuß D. Wolf O. T. (2008). Psychosocial stress induces working memory impairments in an n-back paradigm. Psychoneuroendocrinology 33, 643–653 10.1016/j.psyneuen.2008.02.004 18359168

[B198] Shackman A. J. Maxwell J. S. McMenamin B. W. Greischar L. L. Davidson R. J. (2011a). Stress potentiates early and attenuates late stages of visual processing. J. Neurosci. 31, 1156–1161 10.1523/JNEUROSCI.3384-10.2011 21248140 PMC3037336

[B199] Shackman A. J. Salomons T. V. Slagter H. A. Fox A. S. Winter J. J. Davidson R. J. (2011b). The integration of negative affect, pain and cognitive control in the cingulate cortex. Nat. Rev. Neurosci. 12, 154–167 10.1038/nrn2994 21331082 PMC3044650

[B200] Shackman A. J. McMenamin B. W. Maxwell J. S. Greischar L. L. Davidson R. J. (2009). Right dorsolateral prefrontal cortical activity and behavioral inhibition. Psychol. Sci. 20, 1500–1506 10.1111/j.1467-9280.2009.02476.x 19906125 PMC2858783

[B201] Shackman A. J. Sarinopoulos I. Maxwell J. S. Pizzagalli D. A. Lavric A. Davidson R. J. (2006). Anxiety selectively disrupts visuospatial working memory. Emotion 6, 40 10.1037/1528-3542.6.1.40 16637749

[B202] Shankman S. A. Nelson B. D. Sarapas C. Robison-Andrew E. J. Campbell M. L. Altman S. E. (2012). A psychophysiological investigation of threat and reward sensitivity in individuals with panic disorder and/or major depressive disorder. J. Abnorm. Psychol. [Epub ahead of print]. 10.1037/a0030747 23148783 PMC3694994

[B203] Shechner T. Pelc T. Pine D. Fox N. Bar Haim Y. (2012). Flexible attention deployment in threatening contexts: an instructed fear conditioning study. Emotion 12, 1041–1049 10.1037/a0027072 22390711 PMC3488277

[B204] Sheppes G. Luria R. Fukuda K. Gross J. J. (2013). There's more to anxiety than meets the eye: isolating threat-related attentional engagement and disengagement biases. Emotion. [Epub ahead of print]. 10.1037/a0031236 23356563

[B205] Shin L. M. Bush G. Milad M. R. Lasko N. B. Brohawn K. H. Hughes K. C. (2011). Exaggerated activation of dorsal anterior cingulate cortex during cognitive interference: a monozygotic twin study of posttraumatic stress disorder. Am. J. Psychiatry 168, 979–985 10.1176/appi.ajp.2011.09121812 21724666 PMC3773363

[B206] Shin L. M. Liberzon I. (2009). The neurocircuitry of fear, stress, and anxiety disorders. Neuropsychopharmacology 35, 169–191 10.1038/npp.2009.83 19625997 PMC3055419

[B207] Shin L. M. Wright C. I. Cannistraro P. A. Wedig M. M. McMullin K. Martis B. (2005). A functional magnetic resonance imaging study of amygdala and medial prefrontal cortex responses to overtly presented fearful faces in posttraumatic stress disorder. Arch. Gen. Psychiatry 62, 273–281 10.1001/archpsyc.62.3.273 15753240

[B208] Singh I. L. Dwivedi C. B. Sinha M. M. (1979). Effects of anxiety on vigilance. Percept. Mot. Skills 49, 142–142 503726 10.2466/pms.1979.49.1.142

[B209] Spence J. T. Spence K. W. (1966). The motivational components of manifest anxiety: drive and drive stimuli, in Anxiety and behavior, ed Spielberger C. D. (New York, NY: Academic Press), 291–326

[B210] Spielberger C. D. Gorsuch R. L. Lushene R. E. (1970). The State-Trait Anxiety Inventory. Palo Alto, CA: Consulting Psychologists Press Inc

[B211] Starcke K. Brand M. (2012). Decision making under stress: a selective review. Neurosci. Biobehav. Rev. 36, 1228–1248 10.1016/j.neubiorev.2012.02.003 22342781

[B212] Starcke K. Wolf O. T. Markowitsch H. J. Brand M. (2008). Anticipatory stress influences decision making under explicit risk conditions. Behav. Neurosci. 122, 1352–1360 10.1037/a0013281 19045954

[B213] Ste-Marie C. Gupta R. Derevensky J. L. (2002). Anxiety and social stress related to adolescent gambling behaviour. Int. Gambl. Stud. 2, 123–141 10.1093/aje/kwr017 21467151 PMC3139964

[B214] Stern E. R. Liu Y. Gehring W. J. Lister J. J. Yin G. Zhang J. (2010). Chronic medication does not affect hyperactive error responses in obsessive-compulsive disorder. Psychophysiology 47, 913–920 10.1111/j.1469-8986.2010.00988.x 20230510 PMC3928597

[B215] Stout D. M. Shackman A. J. Larson C. L. (2013). Failure to filter: anxious individuals show inefficient gating of threat from working memory. Front. Hum. Neurosci. 7:58 10.3389/fnhum.2013.00058 23459454 PMC3586709

[B216] Stroop J. R. (1935). Studies of interference in serial verbal reactions. J. Exp. Psychol. 18, 643–662

[B217] Tecce J. T. Happ S. J. (1964). Effects of shock-arousal on a card-sorting test of color-word interference. Percept. Mot. Skills 19, 905–906 14238238 10.2466/pms.1964.19.3.905

[B218] Telzer E. H. Mogg K. Bradley B. P. Mai X. Ernst M. Pine D. S. (2008). Relationship between trait anxiety, prefrontal cortex, and attention bias to angry faces in children and adolescents. Biol. Psychol. 79, 216 10.1016/j.biopsycho.2008.05.004 18599179 PMC2574721

[B219] Thomaes K. Dorrepaal E. Draijer N. De Ruiter M. B. Elzinga B. M. Van Balkom A. J. (2012). Treatment effects on insular and anterior cingulate cortex activation during classic and emotional Stroop interference in child abuse-related complex post-traumatic stress disorder. Psychol. Med. 42, 2337–2349 10.1017/S0033291712000499 22436595

[B220] Vaidyanathan U. Nelson L. D. Patrick C. J. (2012). Clarifying domains of internalizing psychopathology using neurophysiology. Psychol. Med. 42, 447 10.1017/S0033291711001528 21854683 PMC6573029

[B221] Vallar G. Shallice T. (2007). Neuropsychological Impairments of Short-Term Memory. Cambridge: Cambridge University Press

[B222] van den Bos R. Harteveld M. Stoop H. (2009). Stress and decision-making in humans: performance is related to cortisol reactivity, albeit differently in men and women. Psychoneuroendocrinology 34, 1449–1458 10.1016/j.psyneuen.2009.04.016 19497677

[B223] van der Wee N. J. A. Ramsey N. F. Jansma J. M. Denys D. A. Van Megen H. J. G. M. Westenberg H. M. G. (2003). Spatial working memory deficits in obsessive compulsive disorder are associated with excessive engagement of the medial frontal cortex. Neuroimage 20, 2271–2280 10.1016/j.neuroimage.2003.05.001 14683728

[B224] van Tol M. J. van der Wee N. J. A. Demenescu L. R. Nielen M. M. A. Aleman A. Renken R. (2011). Functional MRI correlates of visuospatial planning in out-patient depression and anxiety. Acta Psychiatr. Scand. 124, 273–284 10.1111/j.1600-0447.2011.01702.x 21480834

[B225] Vedhara K. Hyde J. Gilchrist I. Tytherleigh M. Plummer S. (2000). Acute stress, memory, attention and cortisol. Psychoneuroendocrinology 25, 535–549 10.1016/S0306-4530(00)00008-1 10840167

[B226] Vuilleumier P. Armony J. L. Driver J. Dolan R. J. (2001). Effects of attention and emotion on face processing in the human brain: an event-related fMRI study. Neuron 30, 829–841 11430815 10.1016/s0896-6273(01)00328-2

[B227] Vytal K. Cornwell B. Arkin N. Grillon C. (2012). Describing the interplay between anxiety and cognition: from impaired performance under low cognitive load to reduced anxiety under high load. Psychophysiology 49, 842–852 10.1111/j.1469-8986.2012.01358.x 22332819 PMC3345059

[B228] Vytal K. E. Cornwell B. R. Arkin N. E. Letkiewicz A. M. Grillon C. (2013). The complex interaction between anxiety and cognition: insight from spatial and verbal working memory. Front. Hum. Neurosci. 7:93 10.3389/fnhum.2013.00093 23542914 PMC3610083

[B229] Wald I. Degnan K. A. Gorodetsky E. Charney D. S. Fox N. A. Fruchter E. (2013). Attention to threats and combat-related posttraumatic stress symptoms. JAMA Psychiatry 70, 401–408 10.1001/2013.jamapsychiatry.188 23407816 PMC4469781

[B230] Wald I. Lubin G. Holoshitz Y. Muller D. Fruchter E. Pine D. S. (2011). Battlefield-like stress following simulated combat and suppression of attention bias to threat. Psychol. Med. 41, 699–707 10.1017/S0033291710002308 21108868

[B231] Walker R. E. Spence J. T. (1964). Relationship between digit span and anxiety. J. Consult. Psychol. 28, 220–223 14174906 10.1037/h0044190

[B232] Weinberg A. Klein D. N. Hajcak G. (2012). Increased error-related brain activity distinguishes generalized anxiety disorder with and without comorbid major depressive disorder. J. Abnorm. Psychol. 121, 885–896 10.1037/a0028270 22564180 PMC3659236

[B233] Weymar M. Bradley M. M. Hamm A. O. Lang P. J. (2013). When fear forms memories: Threat of shock and brain potentials during encoding and recognition. Cortex 49, 819–826 10.1016/j.cortex.2012.02.012 22483973 PMC3632083

[B234] White M. (1932). Influence of an interpolated electric shock upon recall. J. Exp. Psychol. 15, 752

[B235] Wolf O. T. Schommer N. C. Hellhammer D. H. McEwen B. S. Kirschbaum C. (2001). The relationship between stress induced cortisol levels and memory differs between men and women. Psychoneuroendocrinology 26, 711–720 10.1016/S0306-4530(01)00025-7 11500252

[B236] Wolf O. T. Schommer N. C. Hellhammer D. H. Reischies F. M. Kirschbaum C. (2002). Moderate psychosocial stress appears not to impair recall of words learned 4 weeks prior to stress exposure. Stress 5, 59–64 10.1080/102538902900012332 12171768

[B237] Woodward S. A. McManis M. H. Kagan J. Deldin P. Snidman N. Lewis M. (2001). Infant temperament and the brainstem auditory evoked response in later childhood. Dev. Psychol. 37, 533–538 10.1037/0012-1649.37.4.533 11444488

